# Identification and distribution of novel badnaviral sequences integrated in the genome of cacao (*Theobroma cacao*)

**DOI:** 10.1038/s41598-021-87690-1

**Published:** 2021-04-15

**Authors:** Emmanuelle Muller, Ihsan Ullah, Jim M. Dunwell, Andrew J. Daymond, Megan Richardson, Joël Allainguillaume, Andy Wetten

**Affiliations:** 1 CIRAD, AGAP Institut, 34398 Montpellier, France; 2 AGAP, Univ Montpellier, CIRAD, INRAE, Institut Agro, Montpellier, France; 3 School of Agriculture, Policy and Development, University of Reading, Earley Gate, Reading, RG6 6EU UK; 4 University of the West of England, Frenchay Campus, Coldharbour Lane, Bristol, BS16 1QY UK

**Keywords:** Evolution, Genetics, Molecular biology, Plant sciences

## Abstract

*Theobroma cacao* is one of the most economically important tropical trees, being the source of chocolate. As part of an ongoing study to understand the diversity of the badnavirus complex, responsible for the cacao swollen shoot virus disease in West Africa, evidence was found recently of virus-like sequences in asymptomatic cacao plants. The present study exploited the wealth of genomic resources in this crop, and combined bioinformatic, molecular, and genetic approaches to report for the first time the presence of integrated badnaviral sequences in most of the cacao genetic groups. These sequences, which we propose to name eTcBV for endogenous *T. cacao* bacilliform virus, varied in type with each predominating in a specific genetic group. A diagnostic multiplex PCR method was developed to identify the homozygous or hemizygous condition of one specific insert, which was inherited as a single Mendelian trait. These data suggest that these integration events occurred before or during the species diversification in Central and South America, and prior to its cultivation in other regions. Such evidence of integrated sequences is relevant to the management of cacao quarantine facilities and may also aid novel methods to reduce the impact of such viruses in this crop.

## Introduction

Viral integration within eukaryotic genomes is now a widely recognised phenomenon^1^ and has been described and documented in many species during the last 15 years, thanks to the complete sequencing of many genomes. It occurs in both animals and plants but, unlike animal retroviruses, integration into a plant genome is not an obligatory step in the life cycle of plant viruses. It is proposed that such horizontal gene transfer (HGT) results from illegitimate recombination during the repair of double-stranded DNA breakages^1,2^. Within the plant kingdom, there is evidence that integration of viral sequences occurs in a wide range of families, while the integrated viruses belong mostly to the *Caulimoviridae* family. Such endogenous viral elements (EVEs) belonging to five genera of this family have been characterised in at least 27 plant species from nine different plant families^3–7^.

The distribution and structure of EVEs within plant genomes are diverse and range from short, dispersed, and repetitive viral sequences to longer stretches of near-full length viral genomes. Most EVEs have no deleterious effect on their hosts because they comprise rearranged sequences with inactivating mutations, but in some cases, integrated sequences contain a functional full-length viral genome, which can be activated and lead to systemic infection of the host plant. These latter cases result in infective integration; examples include *Petunia vein clearing virus* (PVCV, genus *Petuvirus*) in petunia^8^, *Tobacco vein clearing virus* (TVCV, genus *Solendovirus*) in tobacco^9^ and *Banana streak virus* (BSV; genus *Badnavirus*) in banana^10–12^.

Cacao (*Theobroma cacao*) is one of the most economically important tropical trees, being vital to the cocoa industry, and providing a livelihood for several million smallholder farmers in the developing world. Particularly in West Africa, growers’ incomes are now threatened by potential reduction in yield caused by susceptibility of the crop to several different viruses of the genus *Badnavirus*^13–15^. However, the presence of most of these viruses has never been described in South and Central America, the geographical origin of the cacao tree.

The diversity of the viruses that infect cacao has been described and can be categorised into at least 11 Badnavirus species^16,17^. These include eight species associated with the commercially important cacao swollen shoot disease (*Cacao swollen shoot Togo A virus*—CSSTAV, *Cacao swollen shoot Togo B virus*—CSSTBV, *Cacao swollen shoot CD virus*—CSSCDV, *Cacao swollen shoot CE virus*—CSSCEV, *Cacao swollen shoot Ghana M virus*—CSSGMV, *Cacao swollen shoot Ghana N virus*—CSSGNV, *Cacao swollen shoot Ghana Q virus*—CSSGQV and the proposed Cacao swollen shoot Ghana T virus—CSSGTV) in West Africa, two species (*Cacao mild mosaic virus*—CaMMV, *Cacao yellow vein banding virus*—CYVBV) described in Trinidad, one of which was also found in Puerto Rico^18^, and one species (*Cacao bacilliform Sri Lanka virus*—CBSLV) from Sri Lanka. One additional cacao badnavirus species [named S species^17^], distinct from all the other known badnaviruses, has been frequently detected in cacao trees in West Africa but the complete genome of this species has not yet been reconstructed.

The search for genetic resistance to *Cacao swollen shoot virus* (CSSV) and other internationally important diseases has stimulated much recent research into the genetic diversity of cacao germplasm available to the breeder. In general terms, the great diversity of cacao genotypes comprises two broad genetic groups “Criollo” and “Forastero” defined on morphological and geographical origins, and a third group “Trinitario” recognized as “Criollo” X “Forastero” hybrids. In recognition of the high genetic diversity within the Forastero grouping, Motamayor et al*.* in 2008 proposed a new classification of *T. cacao* germplasm into ten genetic groups based on Bayesian statistics applied to genotyping data obtained with microsatellite markers^19^. The ten groups, Amelonado, Contamana, Criollo, Curaray, Guiana, Iquitos, Marañon, Nacional, Nanay, and Purús reflect cacao diversity more accurately and this designation is now used for distribution studies of genetic and phenotypic traits. Recently, the genomes of 200 cacao accessions were sequenced and it was shown that along with their assignment to one of the ten genetic population groups, a high number of cacao genotypes correspond to admixed individuals with a differential contribution from several ancestral populations^20^. These analyses provide a more realistic picture of the very complex diversity of cacao as a consequence of genetic diversification and various domestication processes.

As part of an ongoing study of the relationship between CSSV diversity and the cacao host, sequences corresponding to the cacao badnavirus species S have been widely detected in asymptomatic cacao trees, including examples not originating from West Africa. These results raised the possibility of integration of viral sequences from this species in the host genomes. In order to address this question, the present study combined PCR-based analyses with a targeted in silico search for cacao badnavirus S sequences in the wide range of cacao genome sequence data. We show that sections of the genome of this species exist in a variety of integrated forms in the genome of many cacao genotypes. In addition, for the characterized insertion of type VI (Trinitario), a multiplex PCR was developed and used to confirm the Mendelian inheritance of this specific insert, and to characterise the accessions at the International Cocoa Quarantine Centre, the University of Reading, UK (ICQC, R) (http://www.icgd.reading.ac.uk/icqc/).

## Results

### Identification of reverse transcriptase (RT) RNase H badnaviral sequences from asymptomatic cacao plants

**Figure 1:**
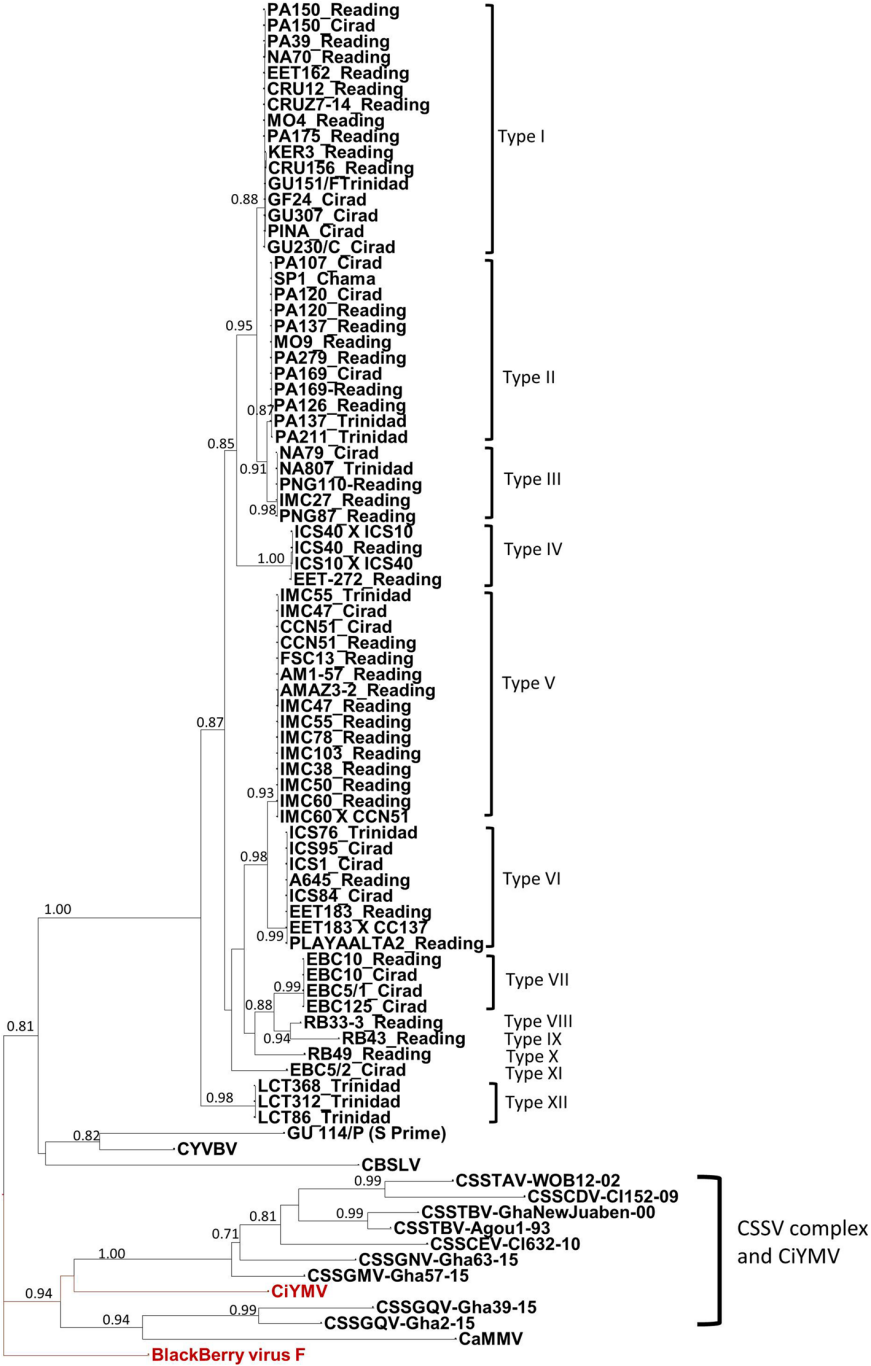
Maximum likelihood phylogenetic tree of badnavirus S sequences based on alignment of the RT RNase H region of open reading frame 3 (ORF3). Numbers on the branches represent the SH-aLRT (approximate Likelihood ratio test) branch supports over 0.7. The Citrus yellow mosaic Virus (CiYMV) (AF347695) and Blackberry virus F (YP009229919) in red colour are used as outgroups along with the other badnavirus infecting cacao trees [CYVBV (KX276640), CaMMV (KX276641), CBSLV (MF642736) and species from the Cacao swollen shoot complex (CSSTAV, AJ781003; CSSTBV, L14546 and AJ608931; CSSCDV, JN606110; CSSCEV, MF642719; CSSGMV, MF642724; CSSGNV, MF642725; CSSGQV, MF642726 and MF642733]. The names of sequences include the name of the cacao clone and the name of the collection from which they were obtained. The 12 different viral types of sequence are identified from I to XII.

In an initial study, a fragment of the expected size of 628 bp was consistently obtained by PCR with the primer pair Badna1deg2/Badna4deg2 (corresponding to RT RNase H region of badnaviruses genomes) from apparently healthy leaf samples collected from the ICQC, R, from the Centre de Coopération Internationale en Recherche Agronomique pour le Développment, France, (CIRAD) collection, and from seedlings grown from germinated seeds. A phylogeny was constructed from the alignment of the amplified fragments (Fig. 1) showing that they correspond to the cacao badnavirus S species, except for one sequence from sample GU 114/P (Guiana group). This sequence is clearly different, with a nucleotide identity of less than 80%, and belongs to another species, provisionally named S prime. Among sequences belonging to cacao badnavirus S, we observed 12 consistent subgroups, each containing a characteristic insert, designated as type I to XII (Fig. 1). Supplemental Table S1 lists the cacao clones that produced PCR products with these primers, along with the viral type obtained. When sequences were amplified from samples of the same clones (CCN 51, EBC 10, PA 120, PA 137, PA 150, IMC 55 and IMC 47) maintained independently in two different collections (ICQC,R, CIRAD or Trinidad), the sequences were identical. Representative samples from the ten cacao genetic groups mentioned above were tested to determine the distribution of such sequences alongside the diversity of cacao genomes according to the previous description^19^. Surprisingly, we observed that this PCR analysis suggested that these badnaviral sequences were present in all cacao diversity groups but with a higher prevalence in the five groups Guiana, Iquitos, Marañon, Nanay, and Purús, and in the group of admixed clones (Table S1 online). By comparison, only two sequences were obtained from the Nacional group and only one sequence from each of the Amelonado, Contamana, Criollo and Curaray groups.

We also observed a correlation between the cacao genomic groups and the type of badnavirus S amplified (Fig. 1 and Table S1 online). All but one of the sequences from samples of the Guiana group contain identical type I sequences. Similarly, all sequences from samples of the Iquitos group contain identical type V sequences.

### Long amplicons and assembly containing sequences of cacao badnavirus S

In order to amplify a more complete genome of the badnavirus S species, inverted abutting primers were designed. These primers, designed in the RT -RNase H region, allowed the amplification of identical 2421 bp amplicons from cacao clones NA 79 and NA 226.

**Figure 2:**
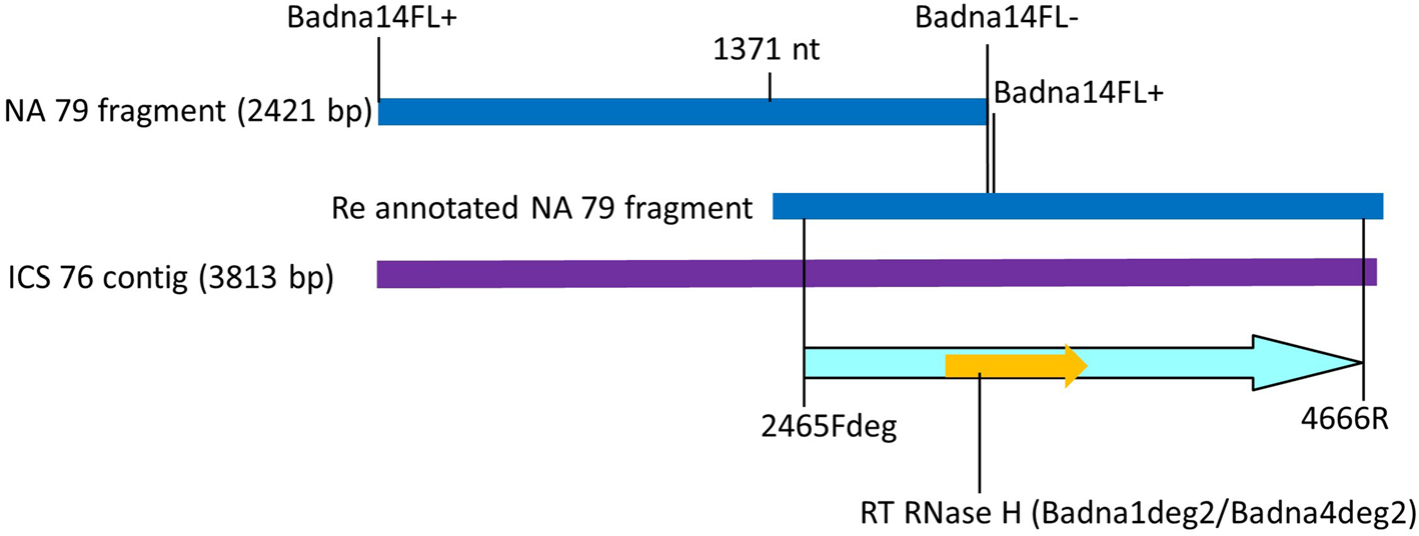
Illustration of design of primers used to amplify S type viral sequences. A fragment of 2421 bp amplified (blue bar) from cacao clone NA 79 with inverted abutting primers Badna14FL + and Badna14FL- was sequenced, re annotated (starting position at 1371) and aligned with a 3813 bp contig constructed from Illumina sequence of cacao clone ICS 76 (purple bar). This alignment was used to design a set of degenerate primers (2465Fdeg and 4666R) for amplification of a ~ 2.2 kb S type viral sequence. Vertical lines indicate locations of primers. Location of RT RNase H region is indicated as a yellow arrow.

From the bioinformatic analysis of the Illumina data from an ICS 76 sample, different sizes of contigs containing badnavirus S sequences were obtained. The sequence of the 2421 bp amplicon amplified from NA 79 and a contig of 3813 nt from ICS 76 were aligned and used to design primers S2465Fdeg /S4666R to amplify a ~ 2.2 kb fragment (Fig. 2). PCR performed with this primer pair amplified a fragment of the expected size from five different groups of cacao clones, namely PA 211 (Marañon), GU 230/C (Guiana), IMC 55 (Iquitos), EBC 5 (Purús) and NA 79 (Nanay), along with the Trinitario clone ICS 95 from an admixed group. BLASTN analysis of these amplicons identified a 1.4 kb region with ~ 60% nucleotide identity in the RT RNase H region of all other badnaviral species.

### In silico screening of whole genome sequencing (WGS) data from cacao

**Figure 3:**
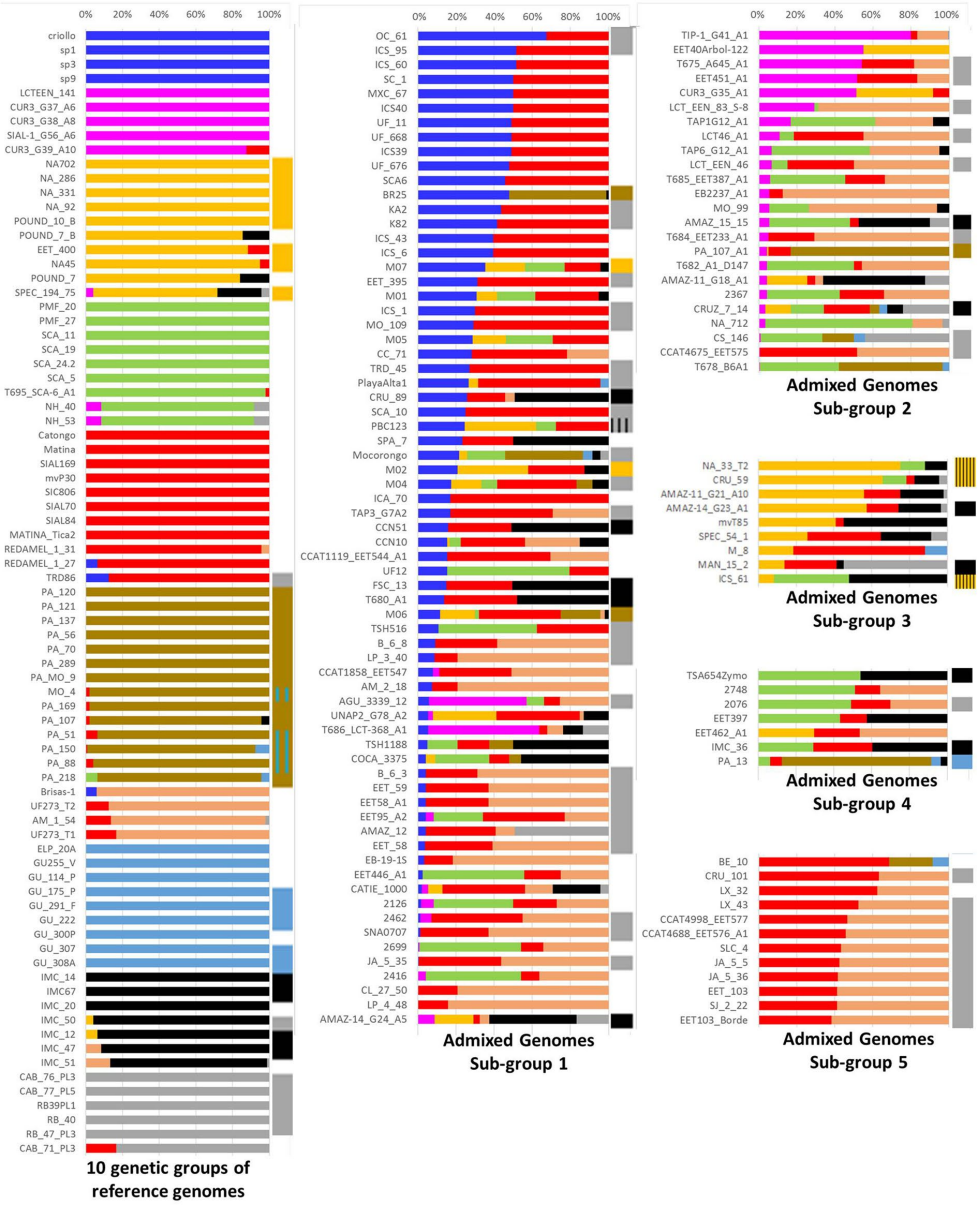
Summary of the bioinformatic search for the presence of the different types of viral sequences in cacao genomes. Bar graph representing population structure in cacao, redrawn from a previous study^20^. The first block on the left side represents 79 clones (with names) grouped into ten distinct genetic groups. The other two blocks represent 121 admixed clones grouped into five sub-groups based on shared proportion of ancestry (horizontal bars). The narrow column on the right side of the individual horizontal bars represents the type of viral sequence found in the clones positive for the viral mapping. The colours correspond to the following groups/**virus types**: Blue (Criollo), Magenta (Curaray), Golden (Nanay/**III**), Green (Contamana), Red (Amelonado), Dark brown (Marañon/**II**)**,** Light brown (Nacional), Light blue (Guiana/**I**), Black (Iquitos/**V)** and Grey (Purús/**VI**). Boxes with hatching patterns represent two viral sequences.

A series of independent WGS datasets were utilised in this analysis. The largest of these datasets was generated in BioProject PRJNA486011, the overall aim of which was to explore the cacao domestication process, and its conclusions provide valuable insights into the evolutionary history of the cacao populations and their population structure^20^. The data generated in this study comprises sequence information from 200 cacao genomes with 5.3–74.5X coverage. We aligned raw data from each of these genomes with viral reference sequences using Bowtie 2 to detect viral sequences. These reference data included sequences of ~ 2.2 kb viral fragments amplified from clones GU 230/C, PA 211, NA 79, IMC 55 and ICS 95, hereafter designated as type I, type II, type III, type V and type VI, respectively (a subset of the total of the 12 different types identified above). Our analysis identified viral sequences in datasets from 103 genomes, out of which a single viral sequence was detected in 95 genomes, and multiple viral sequences were found in eight genomes. Type VI was found to be the most prevalent, detected alone or in combination in 52 and one genomes, respectively. The V, II, III and I types were independently found in 15, 12, 10 and 6 genomes, respectively; these four types were also found in combination in four genomes (Fig. 3, supplemental Table 2). In their study, Cornejo et al*.*^20^ placed 79 reference cacao clones into the ten major genetically distinct groups described above, namely Amelonado, Contamana, Criollo, Curaray, Guiana, Iquitos, Marañon, Nacional, Nanay and Purús. The remaining 121 admixed clones were placed in five arbitrary sub-groups (Fig. 3) based on the major contribution of genetic groups to their mixed ancestry. Our overall results revealed an interesting correlation between genetic grouping of reference clones and mapping of viral sequences. The genomic data search did not detect viral sequence in the reference clones designated as Contamana, Criollo, Curaray, or Nacional. All 11 Amelonado reference clones, except TRD 86, were also found to be free of viral sequences. All 14 Marañon reference clones contained type II viral sequence, whereas 80% of Nanay reference clones had type III sequence. Similarly, type I and V viral sequences were prevalent in Guiana and Iquitos reference clones, respectively. The type VI viral sequence was present in all Purús reference clones except CAB_71_PL3, whereas types V and VI were prevalent in the admixture sub-groups with 70% and 21% representation, respectively (Fig. 3, supplemental Table 2).

The second group of WGS datasets, 31 in total, were downloaded from European Nucleotide Archive (ENA) for BioProject PRJNA558793. In addition, data from the ICS 1 and Catongo Blanco clones were directly downloaded from the NSF (project submitter) website (https://plantscience.psu.edu/research/labs/guiltinan/nsf-plant-genome-research-program). These 33 datasets are notable for their very deep genome coverage (20.8–88.5X). Screening of the datasets detected viral sequences in 23 datasets. The clones used in the study were selected from six of the genetic groups, namely Amelonado, Guiana, Iquitos, Marañon, Nanay and Trinitario, and mapping of viral sequences was found to be correlated to the genetic grouping, as previously found above in BioProject PRJNA486011. Specifically, the clones in the Guiana, Marañon, Nanay and Iquitos genetic groups were mapped with type I, II, III and V viral sequences, the only exception being PA 13 (Marañon), which mapped with type I (Table 1). The Trinitario clone ICS 1 contained the viral type VI as expected. The reliability of the data analysis was confirmed on the basis that there are ten clones common to the PRJNA558793 and PRJNA486011 BioProjects. Common clones positive for the viral mapping, AMAZ 15/15, NA 33, PA 107 and ICS 1 contained the similar viral type V, III + V, II, and VI, respectively in both studies (Table 1). Interestingly, clone PA 13 (Marañon), which mapped to type I in this study also mapped in the analysis above to type I in the dataset from BioProject PRJNA486011, where it is defined as being admixed. It is noteworthy that although the samples for AMAZ 15/15, PA 107 and PA 13 in the two studies were obtained from two different germplasm centres, they produced the same viral type pattern (Table 1).

**Table 1:** Summary of findings from BioProject PRJNA77799^21^ and BioProject PRJNA558793^47^, and comparison with findings from BioProject PRJNA486011^20^, designated as “C”.

PRJNA77799		C	PRJNA558793		C	PRJNA558793			
Clone	Viral type	Viral type	Clone	Viral type	Viral type	Clone	Viral type	Clone	Viral type
*ICS 1*	VI	VI	*AMAZ 12*	–	VI	GU 123V	I	NA 70	III + V
*ICS 6*	–	–	*AMAZ 15/15*	V	V	GU 195V	–	NA 710	III
*ICS 39*	–	–	*Catongo_Blanco*	–	–	GU 257E	–	NA 807	III
*SCA 6*	–	–	*COCA 3370/5*	–	–	IMC 105	V	NA 916	III
Amelonado	–		*ICS 1*	VI	VI	IMC 31	–	OYA 2B	–
Criollo 22	–		*NA 33*	III + V	III + V	IMC 57	V	PA 16	II
EET 64	VI		*PA 107*	II	II	IMC 60	–	PA 279	II + VI
Pentagonum	–		*PA 13*	I	I	KER 1L	I	PA 299	II
Stahel	–		*POUND 7*	–	–	KER 6	I	PA 71	I
*T. grandiflorum*	–		*SPEC 54/1*	–	–	NA 246	II	PA 81	II
			ELP 37A	–		NA 34	III	Pina	I

Italic font (except *T. grandiflora*) indicates clones common to two or more studies.

The third group of WGS datasets, ten in total, were downloaded and analysed from BioProject PRJNA77799^21^ in which the raw read data had 4.2 to 11.0X coverage. This study, which includes six Trinitario clones, and one clone each from Amelonado, Criollo and Forastero, also includes a dataset from the cacao wild relative *T. grandiflorum*. Two Trinitario clones, EET 64 and ICS 1, were mapped with type VI (Table 1). There are four clones common to this study and the 200 genomes study^20^. Both studies confirmed that no viral sequences were found in clones SCA 6, ICS 6 and ICS 39, whereas ICS 1 mapped with type VI virus (Table 1). This latter clone, which is included in all three studies we examined, was mapped to the same viral type, despite the source material being obtained from different germplasm centres (Costa Rica, Puerto Rico, and Trinidad).

We also searched one dataset (MATINA 1/6) from BioProject PRJNA51633^19^, and a long read PacBio dataset of POUND 7, BioProject PRJNA421343^22^, for badnaviral S sequences; however, no viral sequences were identified in either dataset.

### Amplification of virus-plant junction fragment in cacao clone PA 279

**Figure 4:**
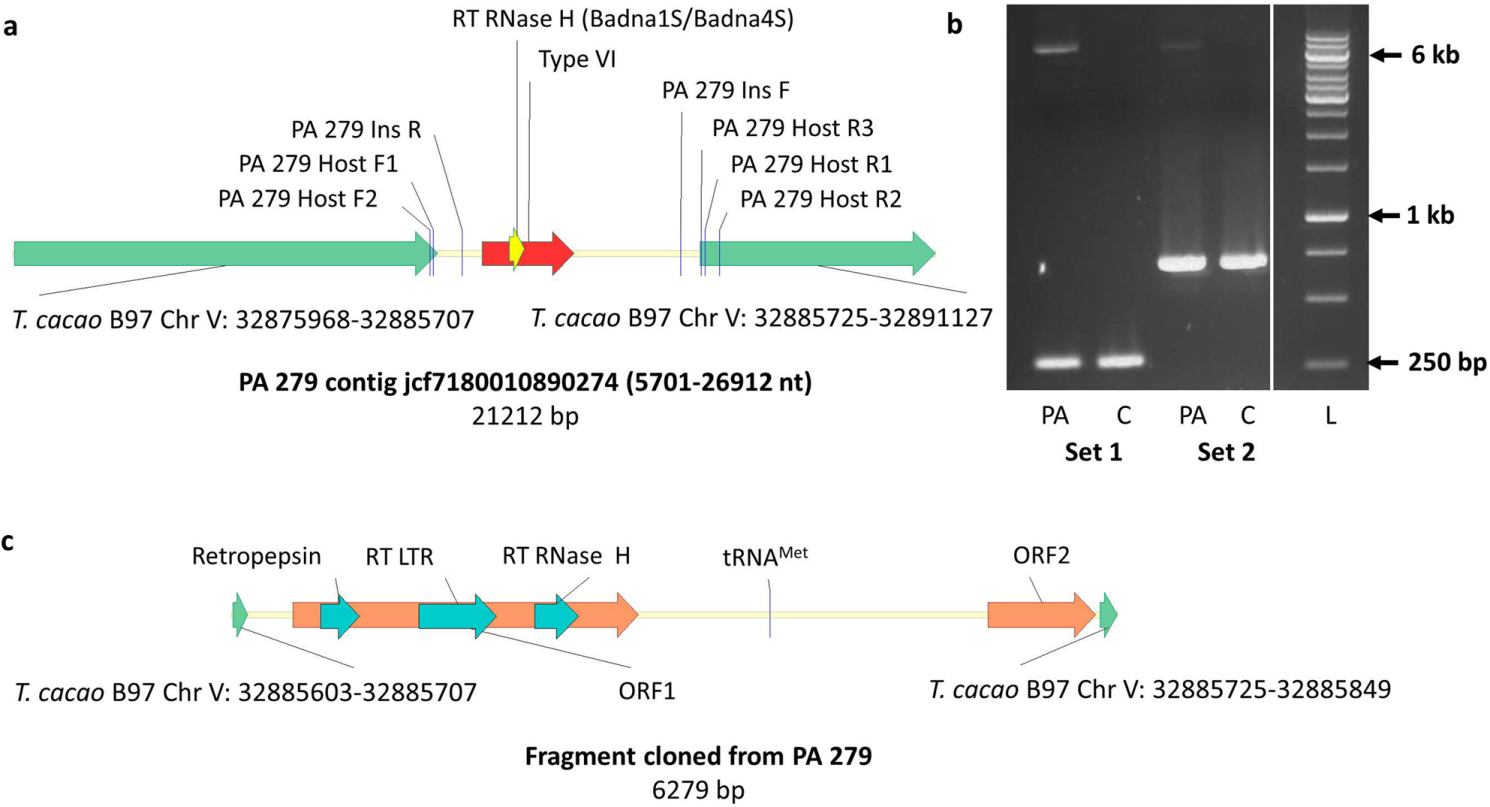
Amplification of type VI viral insertion from cacao clone PA 279. (**a**) Alignment of PA 279 contig jcf7180010890274 and B97 *T. cacao* genome Chromosome V. Green arrows represent genomic region of cacao clone B97 bordering 6050 bp of type VI viral insertion. Vertical lines indicate location of primers including primer set 1 (PA 279 Host F1, PA 279 Host R1) and set 2 (PA 279 Host F2, PA 279 Host R2) used to amplify the viral insert along with the bordering host sequence, and primers used in the multiplex assay to screen cacao germplasm for viral insertion type VI (PA 279 Host F1, PA 279 Ins R, PA 279 Ins F and PA 279 Host R3). (**b**) Amplification of the viral insert along with the bordering host sequence from PA 279 (PA) and Criollo 11 (C) clones. The 243 and 635 bp fragments amplified in both clones with primer set 1 and set 2 represent virus- allele (lacking viral insertion) whereas a fragment between 6 to 7 kb amplified in PA 279 clone in both primer sets represents virus + allele. (**c**) Open reading frames (brown arrows) and conserved domains (light blue arrows) present in the fragment amplified from PA 279 clone harbouring host genome and type VI viral sequence. Full-length gel of section (**b**) is presented in Supplementary Fig. 1.

Based on evidence from the global bioinformatic analysis of the raw genome data from PA 279, a more targeted search for viral sequences was conducted in the preliminary assembly of this clone, downloaded from the NSF website mentioned above. This revealed a type VI sequence in jcf7180010890274, a 51,364 bp contig that contains the viral sequence from position 16,480 to 18,593 nt. A BLASTN search in the B97 *T. cacao* genome (i.e. the reference Criollo *T. cacao* genome available in GenBank) found high similarity of the contig region 1–15,454 and 21,505–51,364 with Chromosome V (GenBank number LT 594,792.1), whereas contig region 15,455–21,504 nt had no similarity with the cacao genome. A BLASTN search of this 6050 nt sequence in the virus database showed similarity with badnaviral sequences corresponding to the RT RNase H region of type VI and the 12 bases tRNA^met^ binding site conserved for every virus of the *Caulimoviridae* family (Fig. 4a). The putative viral region is flanked by B97 *T. cacao* genome Chromosome V, region 32,875,968–32,885,707 nt at the left and 32,885,725- 32,891,127 nt at the right side without a gap.

To further analyse the position of the viral insert, two primer sets were designed flanking the insertion site on B97 *T. cacao* genome Chromosome V. Both sets successfully amplified two bands from PA 279 (accession number RUQ 1119) and one band of expected size (without insertion) from Criollo 11 [CRI] (accession number RUQ 1718). The upper band of around 6.5 kb amplified from PA 279 with primer set I (Fig. 4b) was cloned and sequenced. The complete assembled contig of the clone comprised 6279 bp. The sequencing data revealed 100% similarity with the targeted region of the PA 279 contig jcf7180010890274 mentioned above. An insertion of 6050 bp was found in the genome of PA 279 at Chr V: 32,885,707–32,885,725 nt (B97 *T. cacao* numbering). This insertion site is located in a non-coding region of “uncharacterized” locus LOC18599617. A BLASTN search in the NCBI non-redundant nucleotide database found 98% similarity with B97 Chromosome V at both termini (left terminus from position 1–104 nt, and right terminus from position 6155–6279 nt of the contig). The contig sequence from position 1685–2166 nt showed 100% similarity with Gha68-16 S sequence corresponding to a putative reverse transcriptase protein (ORF3) gene, partial cds (GenBank accession MF784038.1^17^). A 17 nt long deletion was found in the amplified fragment in the host genome immediately after viral sequence insertion site (32,885,708–724 nt, B97 *T. cacao* numbering). The inserted sequence contains two ORFs ≥ 600 nt in length, with the peptide coded by ORF1 consisting of 817 aa and containing a pepsin-like aspartate protease, RT LTR and RT RNase H like conserved domains (Fig. 4c). ORF1 (28–735 aa) shows 59% similarity with Blackberry virus F (GenBank accession YP_009229919.1;^23^) polyprotein. The peptide coded by ORF2 consists of 252 aa and shows similarity to a hypothetical protein in several badnaviral species.

### Screening of germplasm for the type VI insert

Using information generated in the preceding section, a multiplex PCR assay was developed to screen the germplasm collection at ICQC, R. The assay comprises two primer sets, one positioned at the right junction of the insert, the other one at the left junction of the insert. In positive clones (containing the unmodified insert of 6050 nt), the assay amplifies three fragments of 679, 495 and 143 bp, the first two from the two junctions of the insert with host genome, and the third from the host only (in case of a locus hemizygous for the insert). In clones lacking the insert, the assay only amplifies a 143 bp fragment; this also serves as an internal control (Table 2, Fig. 4a). Screening of 342 germplasm accessions from ICQC, R, revealed that 12 and 51 clones contained type VI viral sequence insertion in the homozygous and hemizygous forms, respectively (Table S1 online). The results on positive clones were verified by a second round of tissue sampling, DNA extraction and PCR amplification. Comparison of screening data of multiplex PCR assay and in silico analysis of WGS datasets for the virus insertion type VI showed confirmatory results. All nine clones common to both types of analysis contain the viral sequence except clone AMAZ 12. In the WGS data, the type VI viral sequence was mapped in AMAZ 12 in PRJNA486011; however, we could not detect any viral type in this clone, in either PRJNA558793, or the multiplex PCR study. This inconsistency may be due to accidental mislabelling in one of the source materials.

**Table 2:** PCR primers used in this study.

Specificity	Primer name	Primer sequence^†^	Ta (°C)	Amplicon size (bp)
**RT RNase H region**				
CSSV + S species	Badna1deg2	CCATCCCTTGGACHGCNTTYTGGGT	63	628
	Badna4deg2	TTACATACGGCNCCCCAHCCYTCCAT		
S species	Badna 1S	GATATACTTGTYTTYTCYAACAGCG	59	350
	Badna 4S	GATAAGATTCCARTCRCTDGCCGA		
2.2 kb fragment	S2465Fdeg	ACAGCCCCTRCYRTAAAGGGTCA	63	2202
	S4666R	GCCATAATACGGTAGAGGGAATCATG		
**Inverted abutting primers**				
NA 79	Badna14FL +	GTCAAAAATCTACCTGATATGCGG	57	2421
	Badna14FL-	GTTTGGCTTTGATCTGTCGGATAAG		
**PA 279 junction primers**				
Complete insert and flanking sequence of host	PA 279 Host F1	GGACTATGTTATGCTTGTCCCTT	63	243*
	PA 279 Host R1	ACCAGCCAATGTGAAAATGA		6293**
	PA 279 Host F2	CCATGTGACCATCGTTTCAA	63	635*
	PA 279 Host R2	CGGCTCCTTATTCCAAACAC		6685**
**Genotyping**				
Multiplex	PA 279 Host F1	GGACTATGTTATGCTTGTCCCTTT	63	142*
	PA 279 Ins R	TGGATGCACCAGTATCCAGA		495**
	PA 279 Ins F	TGGCTCCAATGGAAGTTAGC		679**
	PA 279 Host R3	CGGTTGAGAAGAATCCACCT		

^**†**^Letters in primer sequences refer to International Union of Pure and Applied Chemistry (IUPAC) codes for nucleotides.

*expected size in cacao clone PA 279 without viral type VI insertion.

**expected size in cacao clone PA 279 with viral type VI insertion.

### Inheritance study of type VI insert

**Figure 5:**
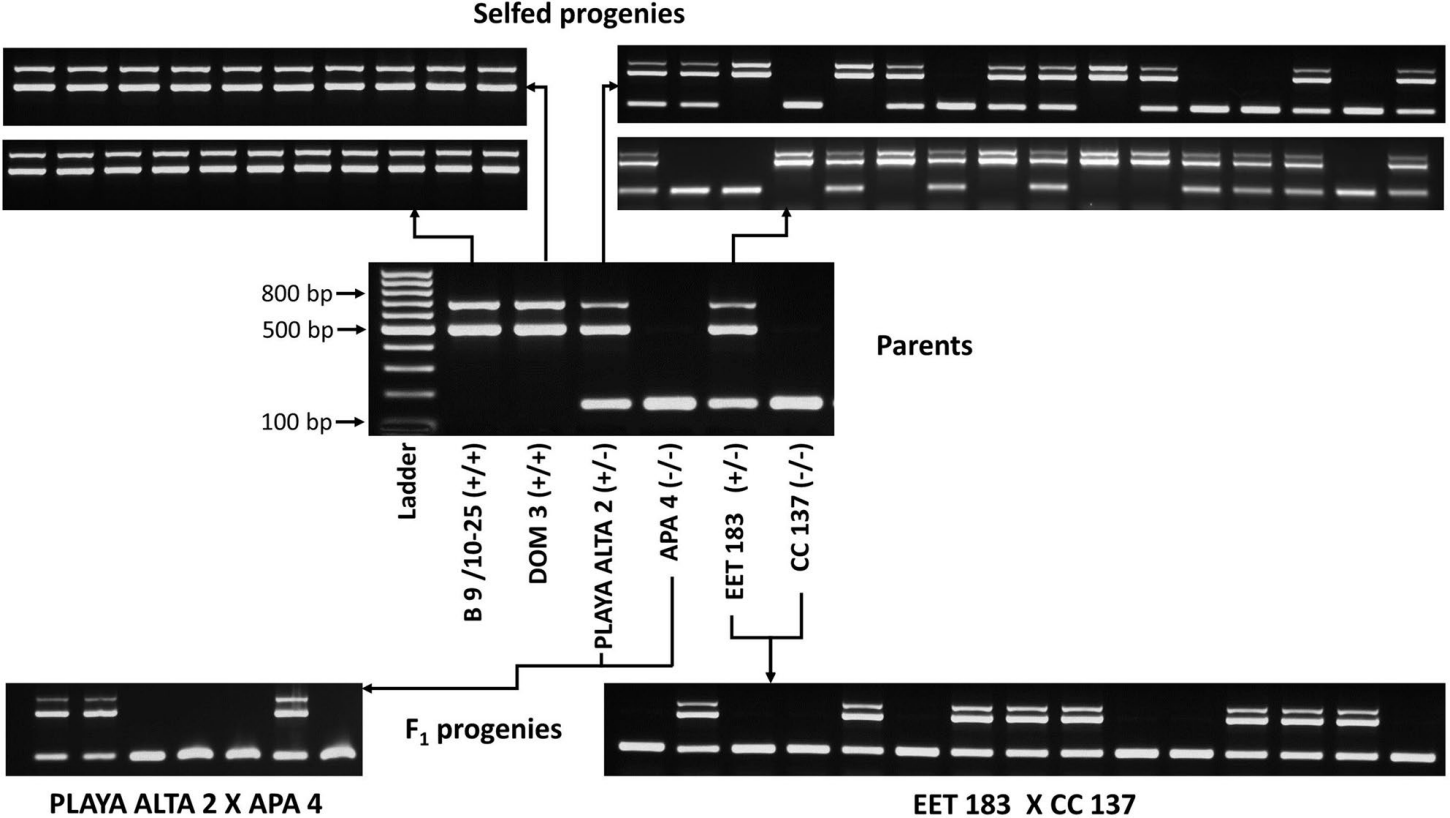
Segregation of viral type VI locus in selfed and crossed progenies of cacao clones. The parents and progenies were genotyped using a multiplex PCR assay including four primers (i.e. PA 279 Host F1, PA 279 Ins R, PA 279 Ins F and PA 279 Host R3).The 495 and 679 bp fragments represent virus + allele, left and right virus/host genome junctions, respectively. The presence of one 142 bp fragment represents virus–allele (−/−), lacking viral insertion. The presence of both 495 and 679 bp fragments in a genotype represents homozygous status (+ / +) of the virus insertion locus, whereas amplification of 142, 495 and 679 bp fragments indicates hemizygous status (+ /−) of the viral insertion locus. Full-length gels are presented in Supplementary Figs. 2–5.

Selfed progenies of two clones each from homozygous and hemizygous type VI virus locus groups (see above), and progenies from two independent crosses between a hemizygous and a negative clone were screened with the multiplex PCR assay to assess the inheritance pattern of the insert. No segregation was found in selfed progenies of clones B 9/10–25 and DOM 3, both homozygous for the virus locus. The progenies of clones EET 183 and PLAYA ALTA 2, both hemizygous for the virus insert, segregated in the classic Mendelian ratio of 1:2:1 (Fig. 5). The progenies of the crosses EET 183 X CC 137 and PLAYA ALTA 2 X APA 4, female parent hemizygous for the virus insert, male parent negative, segregated in the expected ratio of 1:1 (Fig. 5).

## Discussion

Our results clearly demonstrate the first evidence for the presence of badnaviral sequences integrated in cacao genomes, as determined by a range of methodologies employing bioinformatics, molecular analysis and genetic analysis. Following PCR evidence of 12 different viral types belonging to badnavirus species S or species S prime in asymptomatic trees of different genetic groups of cacao, bioinformatic analysis identified a variety of these viral sequences in a large number of cacao clones for which genomic data are available. Correlation between genetic grouping of reference cacao clones and mapping of specific inserts, and discovery of the same viral type in the same clone from multiple studies, confirm integration of the viral sequence in the host genome. The detailed study and sequencing of one specific type VI (Trinitario) insert in the PA 279 cacao clone revealed an insert of 6050 nt in the genome between position 32,885,707 and 328,857,725 of Chromosome V (referring to the numbering on B97 *T. cacao* genome). This approach could be extended to analyse other inserts in the future. We propose to call these integrations eTcBV1 and eTcBV2 for endogenous *Theobroma cacao* bacilliform virus 1 (species S defined as viral type I to XII) and 2 (species S prime) as suggested for the naming of endogenous viruses^5^.

The overall results from the present study extend the list of plants harbouring EVEs, following the most recent description of EVEs in the genomes of *Citrinae*^24^ and red raspberry^25^. Indeed, the cacao genome had already been mentioned as hosting caulimovirid (from the *Caulimoviridae* family) sequences, as reported in 27 plant species, in which sequences of a new proposed viral genus, Florendovirus, had been identified^26^. This particular viral genus is considered to have colonized the genomes of a diversity of species ranging from basal Angiosperms or ANITA grade to monocots and dicots. In 14 plant families, complete genomes were reconstituted, and Florendovirus-like sequences were also found to be present in EST databases^26^.

Unlike these known viral species shown to be present in the genome of their host^10^, in this work we have highlighted sequences belonging to unknown viral species whose existence as episomal particles and/or potential pathogenesis in cacao has not yet been demonstrated.

In summary, and in agreement with the findings for Citrinae^24^ and Musaceae genomes^27^, our results show a wide ‘invasion’ of cacao genomes by badnaviral sequences, significantly so for the cacao genetic groups Guiana, Iquitos, Marañon, Nanay, and Purús, along with the various admixed sub-groups. However, it is possible that we have in fact underestimated the presence of viral inserts, as the absence of PCR products could reflect a variation at the primer locus and does not exclude the presence of other, slightly different, badnaviral integration(s). Similarly, the absence of positive mapping in the four groups Contamana, Criollo, Curaray, and Nacional, and only in one genotype from the Amelonado group, is probably due to the specificity of the sequences used in the present analysis protocol.

As shown in the phylogeny that includes all badnaviral sequences found in cacao genomes (Fig. 1), the presence of PCR positives correspond to viral sequences significantly different from those associated with diseases caused by either CSSV in West Africa, CaMMV or CYVBV in Trinidad or CBSLV in Sri Lanka. Where present, such inserts are probably relics of ancient viral infections that occurred in the South American continent at the period of cacao genetic diversification^19^. As it has been discussed for badnaviral integration in bananas^27^, it would be interesting, not only to extend the search to catalogue the complete range of viral inserts in cacao, but also to uncover both the time-line during which these various inserts took place, and any relationship to the concurrent diversification of the cacao genetic groups. There would seem to be two principal alternative possibilities for this process of integration and diversification. First, all the inserts could have occurred in the early cacao progenitor, followed by selective loss of most inserts during diversification, and consequential presence of a single predominant insert in each group. The second, and perhaps more likely alternative, is that specific inserts occurred in each group at the same time as the diversification into the various cacao groups, and their geographic separation^19^. At present, it would not seem possible definitively to distinguish between the alternatives. In addition, a search for badnavirus sequences in other co-located plant species in the upper Amazon basin may also allow the specific source of these cacao inserts to be identified.

Interestingly, it has been suggested previously that cacao swollen shoot viruses could be transmitted to cacao seeds^28^ but the resultant adult cacao plants from the PCR-positive cacao seedlings never exhibit symptoms of swollen shoot. It could be hypothesized that the high sensitivity associated with the PCR technique could have amplified a virus titre that was too low for an infection to proliferate. PCR fragments were generated in those plants from a conserved region of ORF1 in CSSTBV and CSSTAV species but were sized via capillary electrophoresis and the products were not sequenced. Not all seedlings resulting from pollination were PCR-positive, and the study indicated that the seed transmission of functional, episomal CSSV was not probable. Results presented here on the crosses and progenies (including self progenies) clearly showed that, unlike episomal swollen shoot viruses, these integrated sequences are vertically transmitted to the progenies on a Mendelian basis.

We provide here, for the first time, definitive proof of viral sequence insertion in the cacao genome. This finding helps to explain the positive PCR for presence of virus in symptomless clones. However, the discovery of DNA sequences of the genus *Badnavirus* as integrated sequences in their plant host genome complicates the use of nucleic-acid based diagnostics for badnaviruses in the Cacao swollen shoot species complex that might infect the cacao tree and cause symptoms; this issue has been illustrated by the challenges experienced in reliable detection of banana streak viruses in *Musa* species^11,12,29^. Importantly, the presence of badnaviral inserts in most, if not all, cacao genetic groups, with no evidence of associated symptoms, suggests that there is no risk of spreading any disease by distributing biological material containing these inserted sequences. This issue has been discussed most recently in relation to the evidence for an integrated form of the Rubus Yellow Net Virus in the Red Raspberry genome^25^.

Although this study provides valuable evidence for a variety of badnavirus sequences in the cacao genome, it leaves several important questions to be answered. One of the most obvious is whether the process of integration is completely random or whether there are specific sites into which such integration is most likely to occur. It is known that recombination can occur between badnavirus strains^30,31^, and it may be that the integration process itself occurs preferentially into recombinogenic sequences such as remnants of transposons found widely in plant genomes.

The presence of viral sequences, in whole or in part, that are integrated into host genomes, also raises some interesting evolutionary questions. First, depending on the specific insertion site, one would expect that there may be a positive or negative impact on the phenotype of the plant. For any integrated sequences to be maintained within the genome, it must be assumed that there is a positive impact on the competitiveness of the host plant. If this were not the case, then the integrated sequence would be expected to degenerate over time by the insertion of indels that would disrupt the sequence and then to be eliminated from the cacao genomes. In the present situation in cacao, it would seem that those viral sequences detected to date, though not complete, have maintained their integrity and perhaps therefore they provide some defensive role against the activity of other invasive and potentially damaging viruses by homology-dependent gene silencing^2,6^, as has been suggested for example in yam^32^. This possibility offers the opportunity of manipulating the inserts, for example by gene editing as has been successfully demonstrated in certain genotypes of banana that host infective EVEs^33^. These targeted approaches can be considered as a supplement to the existing projects examining the potential for cross protection provided by mild strains of CSSTBV^34^.

In summary, the data generated in this study add to the growing evidence for the dynamic status of plants (and other) genomes^35^, such as evidence for the role of plant viruses as agents for horizontal gene transfer between species^36^, and the role of such diversity in determining the response of plants to the continuous battle between plants and their pathogens.

## Methods

### Plant material

Fresh leaves of cacao clones from different genetic groups were supplied by CIRAD (Montpellier, France) and ICQC, R and ICG, T (International Cocoa Genebank, Trinidad and Tobago). To confirm whether there was evidence for sexual transmission of possible viral integrations, controlled self- and cross-pollinations were conducted at ICQC, R using clones determined to be positive and negative for the presence of badnaviral sequences. For cross-pollinations, petals and sepals, and then anthers were removed from the recipient flowers before pollination was conducted using the isolated anthers from the pollen donor clone. Following five to six months of pod development, the resultant seeds were collected and germinated. All experimental research conducted on plants complied with relevant institutional, national, and international guidelines and legislation.

### Genomic DNA extraction

Total genomic DNA was isolated from cacao leaves using the Plant DNeasy kit (Qiagen) according to manufacturer’s recommendations. Eighty milligrams of fresh leaves were ground with liquid nitrogen in a microcentrifuge tube in the presence of ceramic beads using a FastPrep-24 Classic (MP Biomedicals) homogenizer. Alternatively, five hundred milligrams of cacao leaves were frozen in liquid nitrogen and ground to a fine powder, which was then mixed with 5 mL of extraction buffer [100 mM tris(hydroxymethyl)aminomethane (Tris)-HCl, pH 8, 1.4 M NaCl, 20 mM ethylenediaminetetraacetic acid (EDTA), 2% w/v mixed alkyltrimethylammonium bromide, 1% w/v PEG6000 and 0.5% w/v Na_2_SO_3_ added freshly]. Samples were incubated at 74 °C for 30 min with 2 mg/mL RNase (Qiagen), extracted twice by 5 mL of chloroform–isoamyl alcohol (24:1) and precipitated with 5 mL of isopropanol at − 20 °C. DNA pellet was rinsed with EtOH and resuspended in 500 μL of sterile distilled deionized water. After quantification, DNA quality was assessed by PCR using the microsatellite mTC 351 primer pair (Table 2)^37^.

### Illumina sequencing of the ICS 76 clone from Trinidad

Extracted DNA from the ICS 76 clone (T3 tree) was sent to Fasteris S.A. (Geneva, Switzerland) for library preparation and sequencing using Illumina HiSeq rapid run technology, which resulted in paired-end reads of 250 bp mean length. Paired-end reads were trimmed using the Cutadapt script^38^ to remove adaptors and filter for quality and were assembled using SPAdes v3.12.0^39^ with k-mers ranging from 21 to 127 (21, 33, 43, 55, 77, 99, 127). All contigs were used to perform a BLAST analysis against a locally created database containing all available sequences representative of the cacao badnavirus S diversity to identify contigs (or scaffolds) containing cacao badnavirus S species.

### PCR amplification

For detection of badnaviral sequences, two primer pairs were designed targeting RT RNase H badnaviral region. The first primer pair, which amplifies a 628 bp fragment, was designed by aligning all badnaviral sequences detected in cacao trees samples from West Africa as previously described^17^. This primer set is potentially able to detect all species associated with Cocoa swollen shoot disease, CSSTAV, CSSTBV, CSSCDV, CSSCEV, CSSGMV, CSSGNV, CSSGQV, CSSGTV, along with the badnaviral species S (not associated with complete genomes). The second primer pair was designed to amplify 366 bp, and specifically detects cacao badnavirus S species. In order to amplify a longer fragment of the genome of the cacao badnavirus species S, inverted abutting primers were designed in the RT RNase H fragment obtained from cacao clone NA 79. Alignment of sequence of the fragment amplified from NA 79 with inverted abutting primers and 3813 nt contig from ICS 76 allowed the design of new primers to amplify a fragment of ~ 2.2 kb fragment containing badnavirus S sequences (Fig. 2).

For detection, amplifications were performed using the Phire Hot Start II DNA Polymerase (Fisher Scientific) according to the manufacturer’s recommendations. For long PCR, the Expand Long Template PCR system (Roche) was used following the manufacturer’s instructions with the Expand Long Template buffer 3, an annealing of 57 °C and an elongation step of 6 min.

Two primer sets were designed flanking the insertion site by using information from alignment of PA 279 contig jcf7180010890274 and B97 *T. cacao* genome Chromosome V to amplify the viral insert along with the bordering host sequence from clone PA 279. The Phusion Green Hot Start II High-Fidelity PCR master mix (Fisher Scientific) was used to amplify the viral insert along with the bordering host sequence from PA 279. The amplicon was cloned with Zero Blunt TOPO PCR Cloning Kit for Sequencing (Fisher Scientific). The information obtained from sequencing of the viral insert amplified from PA 279 was then utilized to design a multiplex PCR assay for detection of the type VI insert in ICQC, R germplasm. Platinum Hot Start PCR Master Mix (Fisher Scientific) was used in the multiplex PCR assay.

The PCR fragments and cloned PCR products were sequenced by Sanger technology (Eurofins Genomics, Germany and Source Bioscience, UK). Information about the primers used in this study including primer sequences, specific targets, and annealing temperatures for PCR are described in Table 2.

### In silico screening of genomic data

The Sequence Read Archive (SRA, https://www.ncbi.nlm.nih.gov/sra) was searched for publicly available Whole Genome Sequence (WGS) and RNA sequence (RNA-Seq) datasets of *T. cacao* (as of April, 2020). The raw read files, of the searched datasets, in FastQ format, were downloaded from the ENA (https://www.ebi.ac.uk/ena/browser/home). The short reads were mapped using Bowtie2 v 2.3.4.1^40^. Minimap2 mapper^41^ was used to align the long (MinION) reads. A database consisting of five badnavirus S sequences (derived from PCR fragments obtained with S2465Fdeg and S4666R primers, Table 2) was used as a reference in both cases. As described above, the five selected types, namely type I, type II, type III, type V and type VI, (a subset of the total of the 12 different clones initially identified) were chosen on the basis of ~ 2.2 kb viral fragments amplified from clones GU 230/C, PA 211, NA 79, IMC 55 and ICS 95, respectively. The mapped reads were compressed, sorted and indexed by Samtools v 1.10^41^. The alignment data were visualised in the Integrative Genomics Viewer (IGV) v.2.4.13^42^. Table 3 lists all the Bioprojects used in this study to search for viral sequences in cacao genomes.

**Table 3:** Whole Genome Sequence (WGS) datasets used in the study.

BioProject	Number of Biosamples	Number of SRA experiments	Read length (bp)	Coverage/size (X)	Number of selected SRA experiments	References
PRJNA486011	200	200	74–202	5.3–74.5	200	^20^
PRJNA558793	31	93	275–300	20.8–88.5	31 + 2*	^47^
PRJNA77799	10	10	60	4.2–11.0	10	^21^
PRJNA421343	1	7	2981–12,905	5.4–14.0	1	^22^
PRJNA51633	8	14	361–869	0.6–2.1	1	^19^

*Dataset directly downloaded from the NSF (project submitter) website (https://plantscience.psu.edu/research/labs/guiltinan/nsf-plant-genome-research-program).

Preliminary assembly of clone PA 279 was downloaded from the Penn State University website http://bigdata.bx.psu.edu/Cacao_NSF_data/. Blast analyses were conducted for the preliminary assembly of PA 279 (https://blast.ncbi.nlm.nih.gov/) using the badnavirus S type VI sequence as the query in order to identify the precise insertion site.

### Phylogenetic analysis

Seaview version 4.0 software was used to analyse the DNA sequences, and these were aligned using the MUSCLE multiple alignment algorithm^43^. Phylogenetic relationships between CSSV sequences were estimated with PhyML (maximum likelihood method^44^) with SH-aLRT (approximate likelihood ratio test^45^) branch supports and phylogenetic trees were visualized with the Darwin 5 program^46^.

## Supplementary Information

Supplementary Information.

## Data Availability

Sequencing data generated in this study were submitted to NCBI GenBank and have Accession Numbers MT993375 and MW009740-MW009807.
