# Supplementary material for: Identification and distribution of novel badnaviral sequences integrated in the genome of cacao (Theobroma cacao)
Source: Sci Rep. 2021 Apr 15;11:8270. doi: 10.1038/s41598-021-87690-1 (PMC8050207; doi:10.1038/s41598-021-87690-1)
Supplement: Supplementary file 1 — Supplementary Information. [file 41598_2021_87690_MOESM1_ESM.pdf]

Identification and Distribution of Novel Badnaviral Sequences Integrated in the Genome of Cacao (*Theobroma cacao*)

Emmanuelle Muller, Ihsan Ullah, Jim M. Dunwell, Andrew J. Daymond, Megan Richardson, Joël Allainguillaume & Andy Wetten

Supplementary Methods

Amplification of virus-plant junction fragment in PA 279 genotype containing the type VI viral insertion

The PCR reaction, which contained 25 µL of Phusion Green Hot Start II High-Fidelity PCR Master Mix, 4 µL each of 5 µM forward and reverse primers, 5 µL of 10 ng/µl DNA template and 12 µL of PCR water, was performed in a thermal cycler (Veriti, Applied Biosystems) programmed to one cycle of 98 °C for 1 min, followed by 35 cycles of 98 °C for 10 s, 63 °C for 10 s and 72 °C for 2 min. Final extension was performed at 72 °C for 10 min. The desired band was eluted from the gel and purified using GeneJET Gel Extraction and DNA Cleanup Micro Kit following manufacturer's instructions. The fragment was cloned into pCR4 Blunt-TOPO Cloning vector using Zero Blunt TOPO PCR Cloning Kit for sequencing following manufacturer's instructions. Colonies were selected for subsequent culturing for expected size by colony PCR. Plasmid DNA was isolated from colony PCR positive cultures using GeneJET Plasmid Miniprep Kit and verified by restriction with *EcoRI* enzyme, which has restriction sites in the cloning vector 10 bases on the left and right of the inserted PCR product. All kits/reagents were purchased from Fisher Scientific, UK. The confirmed positive clone was sequenced by Sanger technology (Source Bioscience, UK). The sequencing data were assembled using SeqMan II sequence analysis software (DNASTAR).

Multiplex PCR assay for screening of germplasm and genotyping of selfed and crossed progenies

The multiplex PCR reaction consisting of 10 µL of Platinum Hot Start PCR Master Mix, 1.5 µL each of 5 µM four primers, 2 µL of 10ng/µL DNA template and 2 µL of PCR water was performed in a thermal cycler (Veriti, Applied Biosystems, UK) programmed to one cycle of 94 °C for 2 min, followed by 30 cycles of 94 °C for 15 s, 63 °C for 15 s and 68 °C for 20 s. The PCR products were resolved on 1.5 % agarose gel and stained with ethidium bromide.

Supplementary Tables

Supplementary Table S1. List of cocoa clones giving specific RT RNase H PCR products with badnaviral primers. Genetic group assignation was conducted according to the higher percentage obtained after classification using 48 SNP markers.

Clone Name	Genetic group	Donor Collection	Country of origin	Sequence type
Catongo	Amelonado	ICG, T	Brazil	ND
VENC 20		CIRAD	Venezuela	ND
SCA 5	Contamana	ICG, T	Peru	ND
B97	Criollo	CIRAD	Belize	ND
SP 1		Chama Station*	Venezuela	II-
LCTEEN 312	Curaray	ICG, T	Ecuador	XII
LCTEEN 57		ICG, T	Ecuador	ND
CRU 156	Guiana	ICQC, R	N/A	I
GF 24		CIRAD	French Guiana	I
GU 114/P		ICG, T	French Guiana	S Prime
GU 151/F		ICG, T	French Guiana	I
GU 195/P		ICG, T	French Guiana	I
GU 230/C		CIRAD	French Guiana	I
GU 307		CIRAD	French Guiana	I
KER 3		ICQC, R	French Guiana	I
PINA		CIRAD	French Guiana	I
AM 1/57	Iquitos	ICQC, R	Peru	V
AMAZ 3/2		ICQC, R	Peru	V
IMC 38		ICQC, R	Peru	V
IMC 47		CIRAD, ICQC, R	Peru	V
IMC 50		ICQC, R	Peru	V
IMC 55		ICG, T, ICQC, R	Peru	V
IMC 60		ICGC, R	Peru	V
IMC 78		ICQC, R	Peru	V
IMC 103		ICQC, R	Peru	V
CRUZ 7/14	Marañon	ICQC, R	Brazil	I
EET 162		ICQC, R	Ecuador	I
MO 4		ICQC, R	Peru	I
MO 9		ICQC, R	Peru	II
PA 107		CIRAD	Peru	II
PA 120		CIRAD, ICQC, R	Peru	II
PA 126		ICQC, R	Peru	II
PA 137		ICG, T, ICQC, R	Peru	II
PA 150		CIRAD, ICQC, R	Peru	I
PA 169		CIRAD, ICQC, R	Peru	II
PA 175		ICQC, R	Peru	I
PA 211		ICG, T	Peru	II
LCTEEN 86	Nacional	ICG, T	Ecuador	XII

IMC 27	Nanay	ICQC, R	Peru	III
NA 79		CIRAD	Peru	III
NA 127		ICG, T	Peru	ND
NA 807		ICG, T	Peru	III
EBC 10/S401	Purús	CIRAD, ICQC, R	Colombia	VII
EBC 125/S9		CIRAD	Colombia	VII
LCTEEN 368		ICG, T	Ecuador	XII
RB 43		ICQC, R	Brazil	IX
RB 49		ICQC, R	Brazil	X
A 645	Admixed	ICQC, R	Ecuador	VI
CCN 51		CIRAD, ICQC, R	Ecuador[1]	V
CRU 12		ICQC, R	N/A	I
EBC 5/S401		CIRAD	Colombia	VII, XI
EET 183		ICG, T	Ecuador	VI
EET 272		ICQC, R	Ecuador	IV
FSC 13		ICQC, R	Brazil	V
ICS 1		CIRAD	Trinidad[1]	VI
ICS 40		ICQC, R	Trinidad[1]	IV
ICS 76		ICG, T	Trinidad[1]	VI
ICS 84		CIRAD	Trinidad[1]	VI
ICS 95		CIRAD	Trinidad[1]	VI
NA 70		ICQC, R	Peru	I
PNG 87		ICQC, R	Venezuela	III
PNG 110		ICQC, R	Papua New Guinea[1]	III
RB 33/3		ICQC, R	Papua New Guinea[1]	VIII

ND: Not determined

CIRAD: Centre de Coopération Internationale en Recherche Agronomique pour le Développement, France

ICG, T: International Cocoa Genebank, Trinidad and Tobago

ICQC, R: International Cocoa Quarantine Centre, Reading, UK

ICS clones are commonly classified as being Trinitarios

[1]Origin of selection

*Chama Station is located in Venezuela

Supplementary Table S2. Summary of findings from BioProject PRJNA486011[20]

A-

Genetic Group	No. of clones	Virus positive clones	Prevalent type of virus
Amelonado	11	1 (TRD86)	VI
Contamana	9	0	-
Criollo	4	0	-
Curaray	5	0	-
Guiana	9	5	I
Iquitos	7	5	V
Marañon	14	14	II
Nacional	4	0	-
Nanay	10	8	III
Purus	6	5	VI
Admixed subgroups	121	65	V: 14X; VI:45X

B-

Virus Type	Found in clones (#)	
	alone	in combination
I	6	4 (I+II)
II	12	4 (I+II)
III	10	3 (III+V)
V	15	3 (III+VI)
VI	52	1 (V+VI)

Supplementary Table S3. Screening of germplasm of International Cocoa Quarantine Centre, Reading (ICQC, R), UK for type VI virus integration. Red and green filled cells represent clones containing insertion in homozygous and hemizygous form, respectively.

Clone Name	Accession	Donor Collection	Locus Status		Pedigree
			PCR	*In Silico*	
A 645 [ECU]	RUQ 1603	INIAP	+/-		
AM 1 /57 [POU]	RUQ 1748	ICG, T	-/-		
AM 1 /8 [POU]	RUQ 469	ICG, T	-/-		
AM 1 /95 [POU]	RUQ 472	ICG, T	-/-		
AMAZ 12	RUQ 334	ICG, T	-/-	+; -	
AMAZ 3 /2	RUQ 91	ICG, T	-/-		AMAZ 3 [CHA] (OP)
AMAZ 5 /2	RUQ 93	ICG, T	-/-		AMAZ 5 [CHA] (OP)
APA 4	RUQ 843	CIRAD	-/-		
APA 5	RUQ 1695	CATIE	-/-		
ARF 12	RUQ 1364	CATIE	-/-		
B 12 /1 [POU]	RUQ 1541	ICG, T	-/-		
B 12 /2 [POU]	RUQ 436	ICG, T	-/-		
B 5 /7 [POU]	RUQ 522	ICG, T	+/-		
B 6 /3 [POU]	RUQ 1595	ICG, T	+/-		
B 9 /10-25 [POU]	RUQ 1527	ICG, T	+/+		
B 9 /10-32 [POU]	RUQ 1702	ICG, T	+/+		
BE 2	RUQ 1733	CATIE	+/-		
BE 5	RUQ 1719	CATIE	-/-		
BE 8	RUQ 1694	CATIE	+/-		
C 40 [TRI]	RUQ 670	BQS	-/-		MXT 55 x MXT 49
C SUL 3	RUQ 1704	CATIE	-/-		
CATIE 1000	RUQ 844	CIRAD	-/-		POUND 12 x CATONGO F1
CC 137	RUQ 1637	CATIE	-/-		UF 12 (OP), UF 12 (OP)
CC 252	RUQ 1506	CATIE	-/-		
CCN 51	RUQ 1736	Nestle	-/-		(ICS 95 x IMC 67) x CCN 1
CL 10 /27	RUQ 1597	ICG, T	-/-		
CL 10 /5	RUQ 1540	ICG, T	-/-		
CL 13 /4	RUQ 1622	ICG, T	-/-		
CL 19 /10	RUQ 905	ICG, T	-/-		
COCA 3348 /44 [CHA]	RUQ 1723	ICG, T	-/-		
COCA 3370 /5 [CHA]	RUQ 137	ICG, T	-/-		
CRINKLE LEAF	RUQ 1700	CIRAD	-/-		
CRIOLLO 11 [CRI]	RUQ 1718	CATIE	-/-		
CRIOLLO 21 [CRI]	RUQ 1682	CATIE	-/-		
CRU 100	RUQ 877	ICG, T	-/-		
CRU 104	RUQ 1585	ICG, T	+/-		
CRU 12	RUQ 1528	ICG, T	+/-		
CRU 124	RUQ 909	ICG, T	-/-		
CRU 126	RUQ 1698	ICG, T	-/-		
MIS_TTOICGT_GU 175 /P	RUQ 880	ICG, T	-/-		
CRU 269	RUQ 1632	ICG, T	-/-		

CRU 56	RUQ 1660	ICG, T	+/-		
CRU 78	RUQ 1629	ICG, T	-/-		
CRU 89	RUQ 1383	ICG, T	-/-		
CRUZ 7 /14	RUQ 1569	ICG, T	-/-		
DOM 14	RUQ 567	BQS	+/-		
DOM 25	RUQ 673	BQS	-/-		
DOM 3	RUQ 543	BQS	+/+		
DOM 4	RUQ 544	BQS	+/+		
EBC 10 /S-401	RUQ 1173	CIRAD	-/-		EBC 10 (OP)
EBC 148	RUQ 1599	ICG, T	-/-		
EBC 5 /S-401	RUQ 1138	CIRAD	-/-		EBC 5 (OP)
EEG 8	RUQ 1726	ICG, T	-/-		
EET 162 [ECU]	RUQ 1692	ICG, T	-/-		Nacional x VEN. Amarillo type
EET 183 [ECU]	RUQ 1640	CATIE	+/-		Nacional (OP), Nacional (OP)
EET 19 [ECU]	RUQ 1630	ICG, T	-/-		EET 19 [ECU] (OP)
EET 233	RUQ 1517	ICG, T	+/-		
EET 272 [ECU]	RUQ 6	Kew	-/-		Forastero Amarillo type (OP)
EET 28 [ECU]	RUQ 1729	ICG, T	+/-		VEN. Amarillo type (OP)
EET 338 [ECU]	RUQ 1749	ICG, T	-/-		VEN. (OP)
EET 387 [ECU]	RUQ 1703	CATIE	-/-		CG x EET 110 [ECU]
EET 399 [ECU]	RUQ 745	ICG, T	-/-		SILECIA 1 [ECU] (OP)
EET 59 [ECU]	RUQ 791	ICG, T	+/-	+	Nacional (OP)
EET 95 [ECU]	RUQ 414	CATIE	+/-		Nacional x VEN. Amarillo type
EQX /J [CHA]	RUQ 107	ICG, T	-/-		
EQX /Z [CHA]	RUQ 109	ICG, T	-/-		
EQX 27 [EQX]	RUQ 857	CIRAD	-/-		EET 59 [ECU] x EET 62 [ECU]
EQX 69 [EQX]	RUQ 100	ICG, T	-/-		COC 3305 [CHA] (OP)
F 303	RUQ 1028	ICA	-/-		
FSC 13	RUQ 1535	ICG, T	-/-		
GDL 3	RUQ 553	BQS	-/-		
GDL 7	RUQ 554	BQS	-/-		
GEBP 123 /A-M [ADI]	RUQ 1648	ICG, T	-/-		IMC 47 x ICS 41
GEBP 15 /A-F [ADI]	RUQ 1641	ICG, T	-/-		NA 399 x SCA 6
GEBP 165 /A-M [ADI]	RUQ 1616	ICG, T	+/-		B 5/3 x ICS 41
GEBP 180 /A-M [ADI]	RUQ 1615	ICG, T	-/-		
GEBP 211 /A-F [ADI]	RUQ 1644	ICG, T	-/-		PA 125 x SCA 6
GEBP 303 /B-M [ADI]	RUQ 1645	ICG, T	+/-		ICS 46 x SLC 18
GEBP 346 /B-F [ADI]	RUQ 1646	ICG, T	-/-		NA 399 x NA 672
GEBP 35 /A-F [ADI]	RUQ 1647	ICG, T	-/-		NA 399 x PA 46
GEBP 37 /A-F [ADI]	RUQ 1626	ICG, T	-/-		M 33 x CL 19/10
GEBP 403 /A-F [ADI]	RUQ 1653	ICG, T	-/-		JA 5/34 x ICS 1
GEBP 426 /A-F [ADI]	RUQ 1658	ICG, T	-/-		ICS 53 x SCA 6
GEBP 428 /A-F [ADI]	RUQ 1654	ICG, T	-/-		ICS 53 x SCA 6
GEBP 450 /A-F [ADI]	RUQ 1651	ICG, T	-/-		M 33 x IMC 2
GEBP 463 /A-F [ADI]	RUQ 1668	ICG, T	-/-		PA 125 x SCA 6
GEBP 509 /A-F [ADI]	RUQ 1675	ICG, T	+/-		POUND 26/C x EQX 3339/12
GEBP 565 /A-F [ADI]	RUQ 1625	ICG, T	-/-		PA 124 x IMC 103

GEBP 571 /A-F [ADI]	RUQ 1670	ICG, T	-/-		PA 124 x IMC 103
GEBP 584 /A-F [ADI]	RUQ 1643	ICG, T	-/-		NA 715 x NA 534
GEBP 585 /A-F [ADI]	RUQ 1659	ICG, T	-/-		NA 715 x NA 534
GEBP 589 /A-F [ADI]	RUQ 1657	ICG, T	-/-		NA 715 x NA 534
GEBP 617 /A-F [ADI]	RUQ 1662	ICG, T	-/-		NA 715 x ICS 40
GEBP 914 /A-F [ADI]	RUQ 1650	ICG, T	-/-		PA 125 x ICS 29
GF 32	RUQ 648	CIRAD	-/-		
GHANA RED	RUQ 1229	Cadbury	-/-		
GU 114 /P	RUQ 813	ICG, T	-/-		GU 114 (OP)
GU 123 /V	RUQ 1068	CIRAD	-/-		GU 123 (OP)
GU 123 /V	RUQ 187	CIRAD	-/-		GU 123 (OP)
GU 125 /C	RUQ 188	CIRAD	-/-		GU 125 (OP)
GU 133 /C	RUQ 190	CIRAD	-/-		GU 133 (OP)
GU 136 /H	RUQ 221	CIRAD	-/-		GU 136 (OP)
GU 144 /C	RUQ 191	CIRAD	-/-		GU 144 (OP)
GU 147 /H	RUQ 222	CIRAD	-/-		GU 147 (OP)
GU 168 /H	RUQ 223	CIRAD	-/-		GU 168 (OP)
GU 171 /C	RUQ 195	CIRAD	-/-		GU 171 (OP)
GU 183 /G	RUQ 1543	CIRAD	-/-		GU 183 (OP)
GU 195 /V	RUQ 1069	CIRAD	-/-		GU 195 (OP)
GU 207 /H	RUQ 225	CIRAD	-/-		GU 207 (OP)
GU 219 /F	RUQ 768	ICG, T	-/-		GU 219 (OP)
GU 221 /C	RUQ 200	CIRAD	-/-		GU 221 (OP)
GU 226 /V	RUQ 1070	CIRAD	-/-		GU 226 (OP)
GU 241 /P	RUQ 1431	ICG, T	-/-		GU 241 (OP)
GU 249 /H	RUQ 228	CIRAD	-/-		GU 249 (OP)
GU 255 /V	RUQ 1549	CIRAD	-/-		GU 255 (OP)
GU 259 /C	RUQ 203	CIRAD	-/-		GU 259 (OP)
GU 261 /P	RUQ 1370	ICG, T	-/-		GU 261 (OP)
GU 263 /V	RUQ 1545	CIRAD	-/-		GU 263 (OP)
GU 265 /P	RUQ 890	ICG, T	-/-		GU 265 (OP)
GU 277 /G	RUQ 771	ICG, T	-/-		GU 277 (OP)
GU 296 /H	RUQ 230	CIRAD	-/-		GU 296 (OP)
GU 310 /P	RUQ 815	ICG, T	-/-		GU 310 (OP)
GU 322 /P	RUQ 816	ICG, T	-/-		GU 322 (OP)
GU 341 /H	RUQ 231	CIRAD	-/-		GU 341 (OP)
ICS 1	RUQ 847	CIRAD	+/-	+	Trinitario type (OP)
ICS 10	RUQ 827	ICG, T	-/-		
ICS 12	RUQ 1688	ICG, T	-/-		NIC (OP)
ICS 15	RUQ 1052	ICG, T	+/-		
ICS 29	RUQ 937	ICG, T	-/-		Criollo (OP)
ICS 35	RUQ 1448	ICG, T	-/-		
ICS 39	RUQ 1089	ICG, T	-/-		NIC (OP)
ICS 40	RUQ 1432	ICG, T	-/-		NIC (OP)
ICS 42	RUQ 1727	ICG, T	-/-		
ICS 43	RUQ 144	ICG, T	-/-		NIC (OP)
ICS 46	RUQ 938	ICG, T	-/-		

ICS 48	RUQ 146	ICG, T	-/-		
ICS 5	RUQ 817	ICG, T	+/-		
ICS 6	RUQ 721	ICG, T	-/-		Trinitario type (OP)
ICS 60	RUQ 959	ICG, T	-/-		NIC (OP)
ICS 63	RUQ 1586	ICG, T	-/-		
ICS 68	RUQ 903	ICG, T	+/-		
ICS 75	RUQ 818	ICG, T	-/-		Trinitario type (OP)
ICS 84	RUQ 1275	CIRAD	+/-		Criollo (OP)
IFC 5 [CIV]	RUQ 848	CIRAD	-/-		
IMC 105	RUQ 863	ICG, T	-/-		
IMC 11	RUQ 8	Kew	-/-		
IMC 14	RUQ 1634	ICG, T	-/-		
IMC 16	RUQ 861	ICG, T	-/-		
IMC 20	RUQ 985	ICG, T	-/-		
IMC 27	RUQ 515	ICG, T	-/-		
IMC 31	RUQ 1449	ICG, T	-/-		
IMC 33	RUQ 1679	ICG, T	-/-		
IMC 38	RUQ 1628	ICG, T	-/-		
IMC 47	RUQ 849	CIRAD	-/-		
IMC 54	RUQ 11	Kew	-/-		
IMC 55	RUQ 836	ICG, T	-/-		
IMC 57	RUQ 1055	ICG, T	-/-		
IMC 58	RUQ 1633	ICG, T	-/-		
IMC 6	RUQ 761	ICG, T	-/-		
IMC 60	RUQ 1617	ICG, T	-/-		
IMC 67	RUQ 1056	ICG, T	-/-		
IMC 71	RUQ 734	ICG, T	-/-		
IMC 78	RUQ 1678	ICG, T	-/-		
IMC 85	RUQ 1666	ICG, T	-/-		
JA 1 /19 [POU]	RUQ 352	ICG, T	+/-		
JA 10 /12 [POU]	RUQ 456	ICG, T	+/-		
JA 5 /5 [POU]	RUQ 1539	ICG, T	+/-	+	
KER 3	RUQ 406	CIRAD	-/-		
KER 6	RUQ 408	CIRAD	-/-		
LCT EEN 15 /S-3	RUQ 685	ICG, T	-/-		
LCT EEN 162 /S-1010	RUQ 686	ICG, T	-/-		
LCT EEN 163 /A	RUQ 178	USDA-SHRS	-/-		
LCT EEN 212 /S-4	RUQ 1665	ICG, T	+/-		LCT EEN 212 (OP)
LCT EEN 261 /S-4	RUQ 1452	ICG, T	-/-		
LCT EEN 302	RUQ 687	ICG, T	-/-		
LCT EEN 341 /S-2	RUQ 1548	INIAP	-/-		
LCT EEN 37 /A	RUQ 148	USDA-SHRS	-/-		
LCT EEN 37 /F	RUQ 153	USDA-SHRS	-/-		
LCT EEN 37 /I	RUQ 156	USDA-SHRS	-/-		
LCT EEN 401	RUQ 1562	INIAP	-/-		
LCT EEN 412	RUQ 1554	INIAP	-/-		
LCT EEN 68 /S-2	RUQ 1594	ICG, T	+/-		

LF 1	RUQ 17	Kew	-/-		Criollo (OP)
LP 3 /15 [POU]	RUQ 1747	ICG, T	-/-		
LP 4 /20 [POU]	RUQ 1676	ICG, T	-/-		
LP 4 /24 [POU]	RUQ 1673	ICG, T	-/-		
LP 4 /32 [POU]	RUQ 883	ICG, T	+/-		
LP 4 /8 [POU]	RUQ 1601	ICG, T	+/-		
LV 17 [POU]	RUQ 1680	ICG, T	-/-		
LV 20 [POU]	RUQ 354	ICG, T	+/-		
LX 31	RUQ 1591	ICG, T	-/-		
LZ 8	RUQ 1231	ICG, T	-/-		
MA 12 [BRA]	RUQ 124	CATIE	-/-		
MA 13 [BRA]	RUQ 1696	ICG, T	-/-		
MAN 15 /2 [BRA]	RUQ 86	ICG, T	-/-		
MAN 15 /60 [BRA]	RUQ 87	ICG, T	-/-		
MAR 9	RUQ 557	BQS	+/+		
MATINA 1 /6	RUQ 1715	USDA-SHRS	-/-		MATINA (OP)
MATINA 1 /7	RUQ 1333	ICG, T	-/-		
MO 109	RUQ 1537	ICG, T	+/-	+	
MO 20	RUQ 1566	ICG, T	-/-		
MO 9	RUQ 1691	ICG, T	-/-		
MOQ 5 /5	RUQ 1669	ICG, T	+/-		
MOQ 6 /19	RUQ 1530	ICG, T	+/-		
MOQ 6 /95	RUQ 1436	ICG, T	-/-		
N 38 [T38]	RUQ 1159	CIRAD	+/-		
NA 111	RUQ 1674	ICG, T	-/-		
NA 149	RUQ 801	ICG, T	-/-		
NA 232	RUQ 1504	ICG, T	-/-		
NA 26	RUQ 1230	ICG, T	-/-		
NA 33	RUQ 1577	ICG, T	-/-		
NA 387	RUQ 1057	ICG, T	-/-		
NA 399	RUQ 1572	ICG, T	-/-		
NA 670	RUQ 1238	ICG, T	-/-		
NA 70	RUQ 1649	ICG, T	-/-		
NA 702	RUQ 1587	ICG, T	-/-		
NA 710	RUQ 1588	ICG, T	-/-		
NA 824	RUQ 1663	ICG, T	-/-		
NA 916	RUQ 1334	ICG, T	-/-		
NAPO 25 [CHA]	RUQ 1547	INIAP	-/-		
PA 120 [PER]	RUQ 852	CIRAD	-/-		
PA 121 [PER]	RUQ 1059	ICG, T	-/-		
PA 124 [PER]	RUQ 1576	ICG, T	-/-		
PA 126 [PER]	RUQ 1721	ICG, T	-/-		
PA 13 [PER]	RUQ 1575	ICG, T	-/-		
PA 136 [PER]	RUQ 1131	ICG, T	-/-		
PA 137 [PER]	RUQ 1081	ICG, T	-/-		
PA 150 [PER]	RUQ 1731	ICG, T	-/-		
PA 156 [PER]	RUQ 1531	ICG, T	-/-		

PA 169 [PER]	RUQ 1479	CATIE	-/-		
PA 175 [PER]	RUQ 37	Kew	-/-		
PA 195 [PER]	RUQ 1642	ICG, T	-/-		
PA 279 [PER]	RUQ 1119	ICG, T	+/-		
PA 299 [PER]	RUQ 1621	ICG, T	-/-		
PA 30 [PER]	RUQ 1635	ICG, T	-/-		
PA 303 [PER]	RUQ 1372	ICG, T	-/-		
PA 39 [PER]	RUQ 1567	ICG, T	-/-		
PA 4 [PER]	RUQ 1058	ICG, T	-/-		
PA 56 [PER]	RUQ 32	Kew	-/-		
PA 67 [PER]	RUQ 590	ICG, T	-/-		
PA 7 [PER]	RUQ 113	MCB	-/-		
PA 70 [PER]	RUQ 33	Kew	-/-		
PA 71 [PER]	RUQ 1564	ICG, T	-/-		
PA 88 [PER]	RUQ 34	Kew	-/-		
PBC 123	RUQ 1499	MCB	+/-	+	Upper Amazon x Trinitario type
PLAYA ALTA 2 [VEN]	RUQ 232	ICG, T	+/-	+	
PMCT 93	RUQ 1249	CATIE	+/-		
PNG 10	RUQ 1314	CIRAD	-/-		
PNG 110	RUQ 1288	CIRAD	-/-		
PNG 138	RUQ 1354	CIRAD	+/-		
PNG 139	RUQ 1355	CIRAD	+/-		
PNG 153	RUQ 1289	CIRAD	+/-		
PNG 155	RUQ 1290	CIRAD	-/-		
PNG 197	RUQ 1291	CIRAD	-/-		
PNG 210	RUQ 1292	CIRAD	+/-		
PNG 215	RUQ 1293	CIRAD	-/-		
PNG 218	RUQ 1294	CIRAD	+/-		
PNG 224	RUQ 1312	CIRAD	-/-		
PNG 296	RUQ 1299	CIRAD	-/-		
PNG 336	RUQ 1321	CIRAD	-/-		
PNG 340	RUQ 1304	CIRAD	+/-		
PNG 360	RUQ 1358	CIRAD	-/-		
PNG 386	RUQ 1306	CIRAD	-/-		
PNG 398	RUQ 1307	CIRAD	-/-		
PNG 414	RUQ 1308	CIRAD	+/-		
PNG 87	RUQ 1287	CIRAD	-/-		
POR 3 [TTO]	RUQ 1062	ICG, T	-/-		
POUND 12 /A	RUQ 1458	ICG, T	-/-		
POUND 16 /B	RUQ 804	ICG, T	-/-		
POUND 18	RUQ 874	ICG, T	-/-		
POUND 7 /B	RUQ 24	Kew	-/-		
RB 33 /3 [BRA]	RUQ 40	Kew	+/+		
RB 37 [BRA]	RUQ 1705	CATIE	+/+		
RB 48 [BRA]	RUQ 1720	CATIE	+/+		
RIM 189 [MEX]	RUQ 310	CATIE	-/-		
RIM 39 [MEX]	RUQ 487	CATIE	-/-		

RUQ 1701	RUQ 1701	CIRAD	-/-		
RUQ 233	RUQ 233	ICG, T	-/-		
SC 1 [UNK]	RUQ 41	Kew	-/-		
SC 20 [COL]	RUQ 690	ICG, T	-/-		
SC 9 [UNK]	RUQ 44	Kew	-/-		
SCA 11	RUQ 1686	ICG, T	-/-		
SCA 12	RUQ 1689	ICG, T	-/-		
SCA 6	RUQ 234	ICG, T	-/-		
SCA 9	RUQ 1064	ICG, T	-/-		
SHRS-01	RUQ 1708	USDA-SHRS	-/-		
SHRS-02	RUQ 1712	USDA-SHRS	-/-		
SHRS-04	RUQ 1709	USDA-SHRS	-/-		
SHRS-05	RUQ 1711	USDA-SHRS	-/-		
SHRS-06	RUQ 1714	USDA-SHRS	+/-		
SHRS-07	RUQ 1716	USDA-SHRS	-/-		
SHRS-08	RUQ 1713	USDA-SHRS	-/-		
SHRS-09	RUQ 1710	USDA-SHRS	-/-		
SHRS-37	RUQ 1734	USDA-SHRS	-/-		
SIAL 339	RUQ 48	Kew	-/-		
SILECIA 5 [ECU]	RUQ 1349	INIAP	-/-		
SJ 1 /40 [POU]	RUQ 1438	ICG, T	-/-		
SLA 16	RUQ 1092	ICG, T	-/-		
SLC 18	RUQ 1134	ICG, T	+/-		
SLC 19	RUQ 1664	ICG, T	+/-		
SNK 413	RUQ 854	CIRAD	-/-		
SPA 16 [COL]	RUQ 691	ICG, T	-/-		
SPA 7 [COL]	RUQ 1552	ICG, T	-/-		
SPA 9 [COL]	RUQ 235	ICG, T	-/-		
SPEC 160 /9	RUQ 51	Kew	-/-		SPEC 160 (OP)
SPEC 194 /15	RUQ 1439	ICG, T	-/-		SPEC 194 (OP)
SPEC 41 /6-18	RUQ 1325	ICG, T	-/-		SPEC 41 (OP)
SPEC 54 /1	RUQ 50	Kew	-/-		SPEC 54 (OP)
T 85 /799 [POS]	RUQ 855	CIRAD	-/-		
TAP 12 [CHA]	RUQ 1725	ICG, T	-/-		
TARS 1	RUQ 1387	USDA-TARS	-/-		UF 668 x POUND 7 [POU]
TARS 14	RUQ 1389	USDA-TARS	-/-		SCA 6 x EET 62 [ECU]
TARS 23	RUQ 1446	USDA-TARS	-/-		UF 668 x POUND 7 [POU]
TARS 31	RUQ 1394	USDA-TARS	-/-		SCA 6 x EET 62 [ECU]
TARS 34	RUQ 1513	USDA-TARS	-/-		UF 668 x POUND 7 [POU]
TRD 109	RUQ 1234	ICG, T	+/+		
TRD 32	RUQ 1534	ICG, T	-/-		
TRD 44	RUQ 1235	ICG, T	-/-		
TRD 45	RUQ 1441	ICG, T	+/+	+	
TRD 85	RUQ 1442	ICG, T	-/-		
TSA 654	RUQ 1029	CEPLAC/CEPEC	-/-		SCA 6 x IMC 67
TSA 656	RUQ 1016	CEPLAC/CEPEC	-/-		SCA 6 x IMC 67
TSAN 792	RUQ 1031	CEPLAC/CEPEC	-/-		TSA 641 x op

TSH 516	RUQ 1018	CEPLAC/CEPEC	+/+	+	ICS 1 x SCA 6
TSH 565	RUQ 1741	Nestle, France	+/+		ICS 1 x SCA 6
TSH 774	RUQ 1020	CEPLAC/CEPEC	+/-		
U 26 [PER]	RUQ 1496	Peru	-/-		
U 45 [PER]	RUQ 1495	Peru	-/-		
U 70 [PER]	RUQ 1488	Peru	-/-		
UF 168	RUQ 1677	ICG, T	-/-		
UF 273	RUQ 1707	CATIE	-/-		
UF 613	RUQ 1573	ICG, T	+/-		Trinitario type (OP)
UF 667	RUQ 55	Kew	-/-		Trinitario type (OP)
UF 676	RUQ 56	Kew	-/-		Trinitario MORADO type (OP)
UF 712	RUQ 1683	CATIE	-/-		Nacional (OP)
UNAP 2 [CHA]	RUQ 1604	INIAP	-/-		
UPA 134	RUQ 1172	CEPLAC/CEPEC	-/-		
VB 547	RUQ 1035	CEPLAC/CEPEC	+/-		
VB 663	RUQ 1036	CEPLAC/CEPEC	-/-		
VB 681	RUQ 1037	CEPLAC/CEPEC	-/-		
WA 40 [DR]	RUQ 1283	CIRAD	-/-		

**+/+**	Homozygous insertion (Highlighted Red)
**+/-**	Hemizygous Insertion (Highlighted Green)
**-/-**	Homozygous wild type
+;-	Different database accessions showed presence and absence of viral sequence

**OP**	Open pollinated
**BQS**	Barbados Quarantine Station, Barbados
**CATIE**	Centro Agronómico Tropical de Investigación y Enseñanza, Costa Rica
**CEPLAC/CEPEC**	Comissão Executiva do Plano da Lavoura Cacaueira/Centro de Pesquisas do Cacau, Brazil
**CIRAD**	Centre de Coopération Internationale en Recherche Agronomique pour le Développment, France
**ICG, T**	International Cocoa Genebank, Trinidad, Trinidad and Tobago
**INIAP**	Instituto Nacional de Investigaciones Agropecuarias, Ecuador
**KEW**	Royal Botanic Gardens, Kew, UK
**USDA-SHRS**	United States Department of Agriculture, Subtropical Horticultural Research Station, Miami
**USDA-PR**	United States Department of Agriculture, Tropical Agriculture Research Station, Puerto Rico

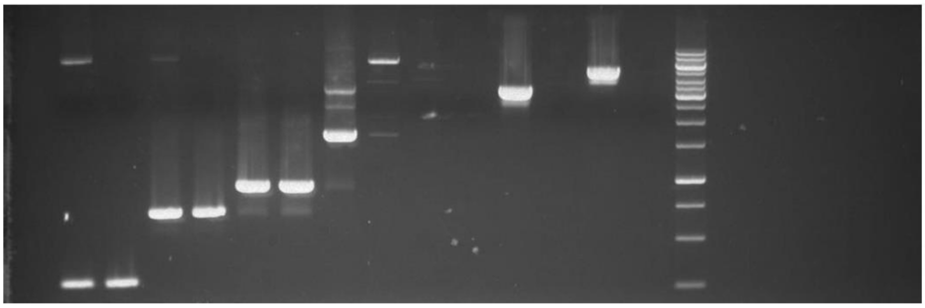

Supplementary Figure S1. Amplification of type VI viral insertion from cacao clone PA 279. **A**- Alignment of PA 279 contig jcf7180010890274 and B97 *T. cacao* genome Chromosome V. Green arrows represent genomic region of cacao clone B97 bordering 6050 bp of type VI viral insertion. Vertical lines indicate location of primers including primer set 1 (PA 279 Host F1, PA 279 Host R1) and set 2 (PA 279 Host F2, PA 279 Host R2) used to amplify the viral insert along with the bordering host sequence, and primers used in the multiplex assay to screen cacao germplasm for viral insertion type VI (PA 279 Host F1, PA 279 Ins R, PA 279 Ins F and PA 279 Host R3). **B**- Amplification of the viral insert along with the bordering host sequence from PA 279 (PA) and Criollo 11 (C) clones. The 243 and 635 bp fragments amplified in both clones with primer set 1 and set 2 represent virus- allele (lacking viral insertion) whereas a fragment between 6 to 7 kb amplified in PA 279 clone in both primer sets represents virus+ allele. **C**- Open reading frames (brown arrows) and conserved domains (light blue arrows) present in the fragment amplified from PA 279 clone harbouring host genome and type VI viral sequence. Lanes 1-4 and ladder from section **B** are given in Figure 4 of main text.

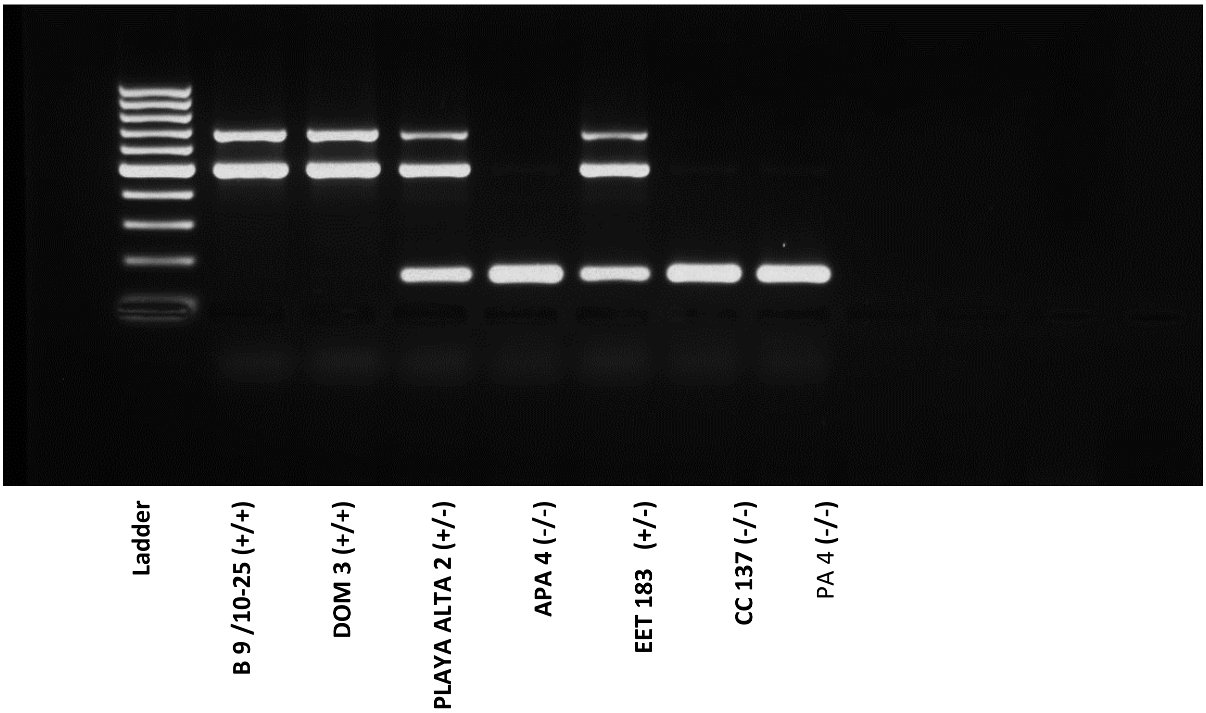

Supplementary Figure S2. Status of viral type VI locus in selected cacao clones. The cacao clones selected as parents were genotyped using a multiplex PCR assay including four primers (i.e. PA 279 Host F1, PA 279 Ins R, PA 279 Ins F and PA 279 Host R3). The 495 and 679 bp fragments represent virus+ allele, left and right virus/host genome junctions, respectively. The presence of one 142 bp fragment represents virus– allele (-/-), lacking viral insertion. The presence of both 495 and 679 bp fragments in a genotype represents homozygous status (+/+) of the virus insertion locus, whereas amplification of 142, 495 and 679 bp fragments indicates hemizygous status (+/-) of the viral insertion locus.

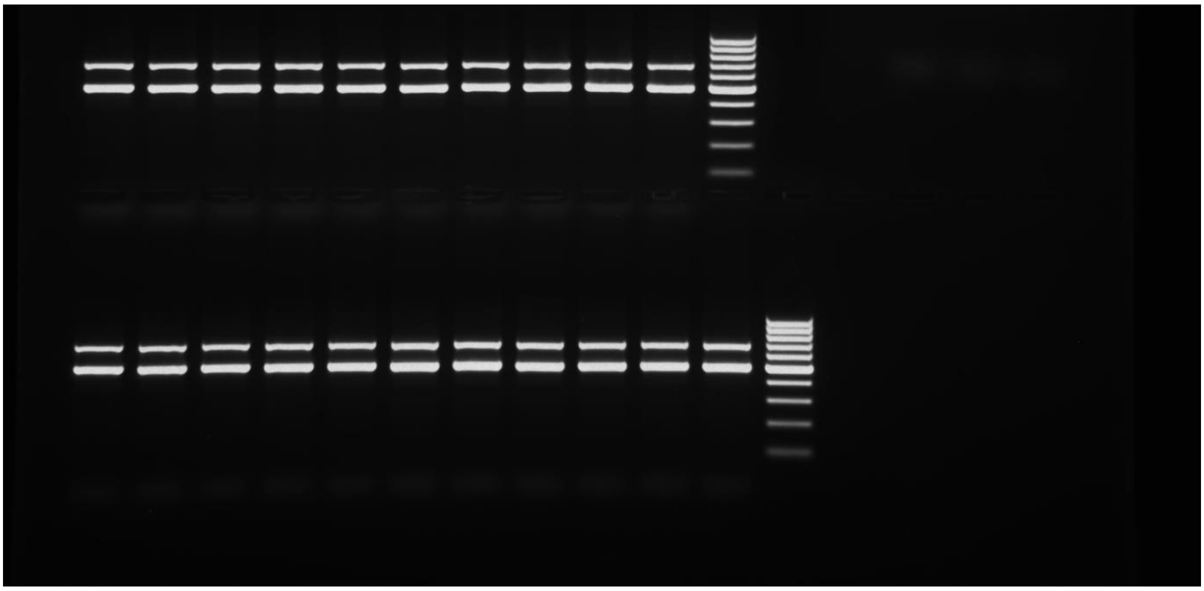

Supplementary Figure S3. Segregation of viral type VI locus in progenies. The selfed progenies of the homozygous cacao clone B 9 /10-25 (upper comb) and DOM 3 (lower comb) were genotyped using a multiplex PCR assay including four primers (i.e. PA 279 Host F1, PA 279 Ins R, PA 279 Ins F and PA 279 Host R3). The presence of 495 and 679 bp fragments in a genotype represents homozygous status of the virus insertion locus.

a

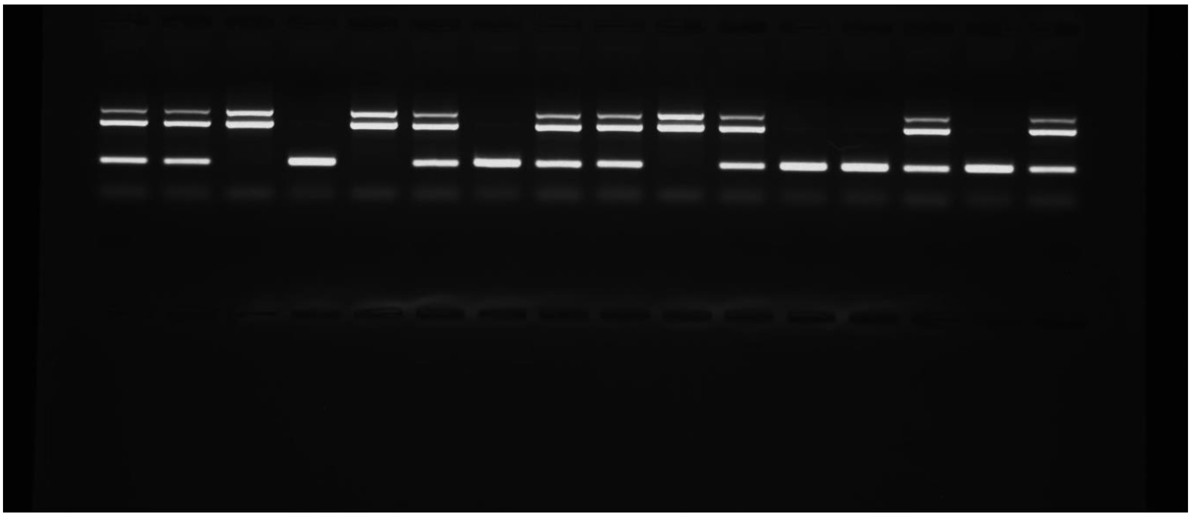

b

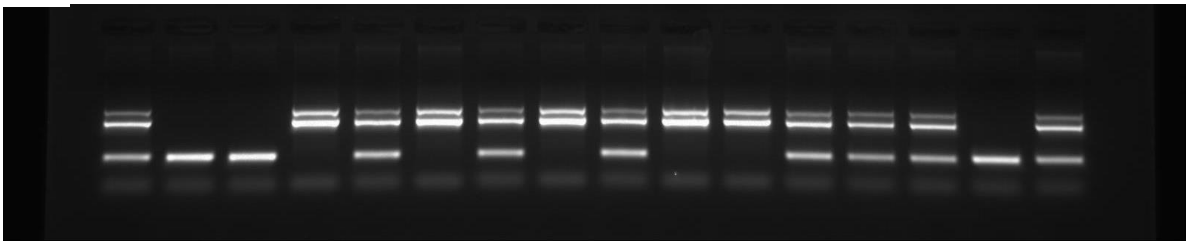

Supplementary Figure S4. Segregation of viral type VI locus in progenies. The selfed progenies of the hemizygous cacao clone PLAYA ALTA 2 (**a**) and EET 183 (**b**) were genotyped using a multiplex PCR assay including four primers (i.e. PA 279 Host F1, PA 279 Ins R, PA 279 Ins F and PA 279 Host R3). 495 and 679 bp fragments represent virus+ allele, left and right virus/host genome junctions, respectively. The presence of one 142 bp fragment represents virus– allele, lacking viral insertion. The presence of both 495 and 679 bp fragments in a genotype represents homozygous status of the virus insertion locus, whereas amplification of 142, 495 and 679 bp fragments indicates hemizygous status of the viral insertion locus.

a

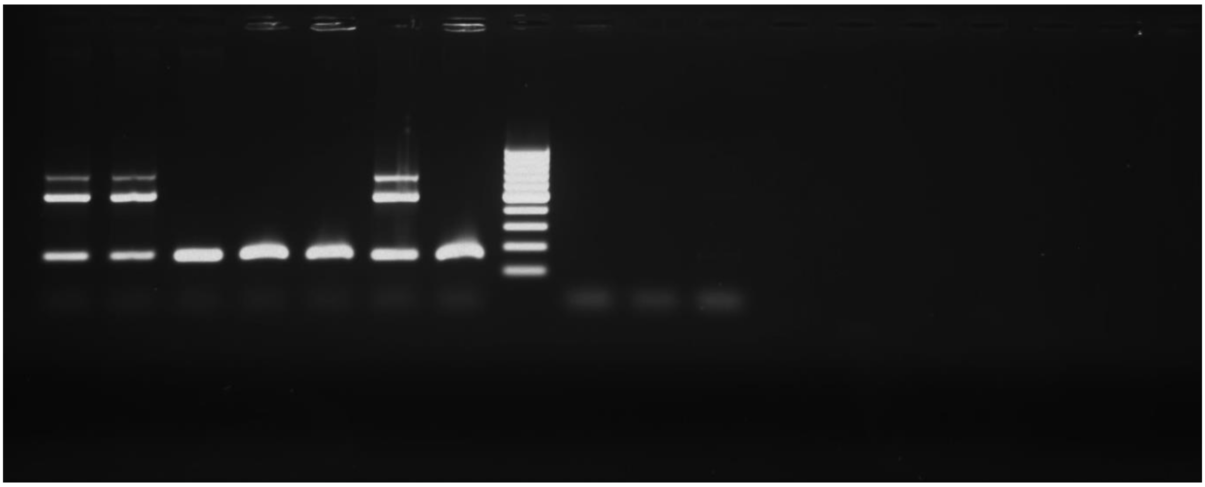

b

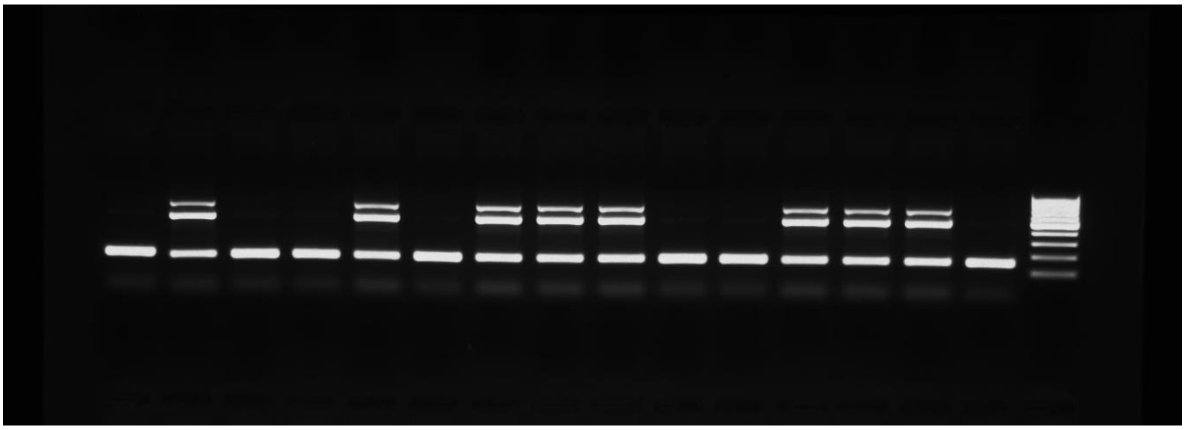

Supplementary Figure S5. Segregation of viral type VI locus in progenies. The progenies of the cross between hemizygous cacao clone PLAYA ALTA 2 and virus locus- clone APA 4 (**a**) and hemizygous cacao clone EET 183 and virus locus- clone CC 137 (**b**) were genotyped using a multiplex PCR assay including four primers (i.e. PA 279 Host F1, PA 279 Ins R, PA 279 Ins F and PA 279 Host R3). 495 and 679 bp fragments represent virus+ allele, left and right virus/host genome junctions, respectively. The presence of one 142 bp fragment represents virus– allele, lacking viral insertion. The amplification of 142, 495 and 679 bp fragments indicates hemizygous status of the viral insertion locus.
